# Luminal androgen receptor breast cancer subtype and investigation of the microenvironment and neoadjuvant chemotherapy response

**DOI:** 10.1093/narcan/zcac018

**Published:** 2022-06-17

**Authors:** Kevin J Thompson, Roberto A Leon-Ferre, Jason P Sinnwell, David M Zahrieh, Vera J Suman, Filho Otto Metzger, Sarah Asad, Daniel G Stover, Lisa Carey, William M Sikov, James N Ingle, Minetta C Liu, Jodi M Carter, Eric W Klee, Richard M Weinshilboum, Judy C Boughey, Liewei Wang, Fergus J Couch, Matthew P Goetz, Krishna R Kalari

**Affiliations:** Mayo Clinic, Department of Quantitative Health Sciences, Rochester, MN, USA; Mayo Clinic, Department of Oncology, Rochester, MN, USA; Mayo Clinic, Department of Quantitative Health Sciences, Rochester, MN, USA; Mayo Clinic, Department of Quantitative Health Sciences, Rochester, MN, USA; Mayo Clinic, Department of Quantitative Health Sciences, Rochester, MN, USA; Harvard Medical School, Department of Medical Oncology, Boston, MA, USA; The Ohio State University Wexner Medical Center, Molecular, Cellular, and Developmental Biology, Columbus, OH, USA; The Ohio State University Wexner Medical Center, Molecular, Cellular, and Developmental Biology, Columbus, OH, USA; University of North Carolina at Chapel Hill School of Medicine, Medical Science, Chapel Hill, NC, USA; Warren Alpert Medical School of Brown University, Department of Medicine Women, Providence, RI, USA; Infants Hospital of Rhode Island, Department of Obstetrics & Gynecology, Providence, RI, USA; Mayo Clinic, Department of Oncology, Rochester, MN, USA; Mayo Clinic, Department of Oncology, Rochester, MN, USA; Mayo Clinic, Department of Laboratory Medicine and Pathology, Rochester, MN, USA; Mayo Clinic, Department of Laboratory Medicine and Pathology, Rochester, MN, USA; Mayo Clinic, Department of Quantitative Health Sciences, Rochester, MN, USA; Mayo Clinic, Department of Laboratory Medicine and Pathology, Rochester, MN, USA; Mayo Clinic, Department of Molecular Pharmacology and Experimental Therapeutics, Rochester, MN, USA; Mayo Clinic, Department of Surgery, Rochester, MN, USA; Mayo Clinic, Department of Molecular Pharmacology and Experimental Therapeutics, Rochester, MN, USA; Mayo Clinic, Department of Laboratory Medicine and Pathology, Rochester, MN, USA; Mayo Clinic, Department of Oncology, Rochester, MN, USA; Mayo Clinic, Department of Molecular Pharmacology and Experimental Therapeutics, Rochester, MN, USA; Mayo Clinic, Department of Quantitative Health Sciences, Rochester, MN, USA

## Abstract

Triple-negative breast cancer (TNBC) is the most aggressive breast cancer subtype with low overall survival rates and high molecular heterogeneity; therefore, few targeted therapies are available. The luminal androgen receptor (LAR) is the most consistently identified TNBC subtype, but the clinical utility has yet to be established. Here, we constructed a novel genomic classifier, LAR-Sig, that distinguishes the LAR subtype from other TNBC subtypes and provide evidence that it is a clinically distinct disease. A meta-analysis of seven TNBC datasets (*n* = 1086 samples) from neoadjuvant clinical trials demonstrated that LAR patients have significantly reduced response (pCR) rates than non-LAR TNBC patients (odds ratio = 2.11, 95% CI: 1.33, 2.89). Moreover, deconvolution of the tumor microenvironment confirmed an enrichment of luminal epithelium corresponding with a decrease in basal and myoepithelium in LAR TNBC tumors. Increased immunosuppression in LAR patients may lead to a decreased presence of cycling T-cells and plasma cells. While, an increased presence of myofibroblast-like cancer-associated cells may impede drug delivery and treatment. In summary, the lower levels of tumor infiltrating lymphocytes (TILs), reduced immune activity in the micro-environment, and lower pCR rates after NAC, suggest that new therapeutic strategies for the LAR TNBC subtype need to be developed.

## Graphical Abstract

**Graphical Abstract:**
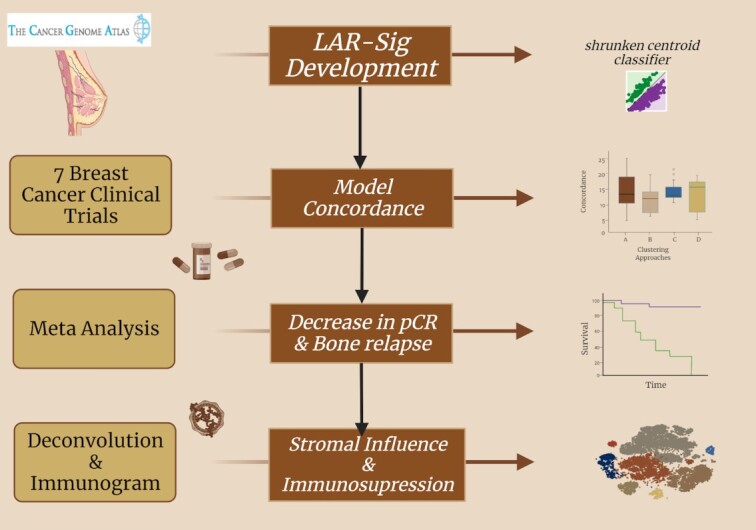
An analysis of luminal androgen receptor triple negative breast cancers: immune activity, tumor microenvironment and neoadjuvant responses. Created with BioRender.com.

## INTRODUCTION

Triple-negative breast cancer (TNBC) is defined by a lack of expression of the estrogen receptor (ER) and progesterone receptor (PR) and lack of overexpression of the human epidermal growth factor receptor 2 (HER2, ERBB2). Patients with TNBC are often diagnosed at younger age and experience a higher risk of relapse and mortality rates than patients with other breast cancer subtypes (1). While patients with TNBC have been managed clinically as a single disease for many years, there is increased recognition that TNBC is not a single entity but rather a group of diseases, and targeted therapies for subsets of TNBC patients with tumors expressing patient-specific biomarkers have recently been developed and approved by regulatory agencies (2–4). While distinct TNBC subtypes have been identified by gene expression profiling (5–12), there still exists an urgent clinical need to identify subtype-specific TNBC treatment regimens (13).

The intrinsic gene signature (PAM50)—the most widely accepted breast cancer molecular classification system—characterizes most TNBC as basal-like in origin. This signature adheres to a two-cell origin model, where TNBC is presumed to arise from the basal layer of myoepithelial cells (10,13,14). However, approximately 25% of TNBCs do not classify as basal-like with PAM50, supporting the notion of its inherent diversity (10). Studies attempting to sub-classify TNBC have identified 3–6 subtypes. The initial report in 2011 by Lehmann *et al.* identified six molecular subtypes (basal-like 1 (BL1), basal-like 2 (BL2), immunomodulatory (IM), mesenchymal (M), mesenchymal stem-like (MSL) and luminal androgen receptor (LAR)); and provided preliminary evidence that the individual subtypes may lend themselves to different therapeutic strategies (6). Subsequent efforts demonstrated strong negative correlations between the IMM and MSL centroids. A refined classification schema replaces the mesenchymal stem-like (MSL) and immunomodulatory (IMM) subtypes with the next highest correlated centroid, as these specimens represent samples enriched in mesenchymal stromal cells and tumor-infiltrating lymphocytes (TILs), respectively (8,15). Another study of a TNBC microarray dataset by Jezequel *et al.* similarly observed a cluster of samples enriched in immunological cells (5,16). The mesenchymal stem-like and basal-like immune active sub-groups identified by Burnstein *et al.* presumably represent samples with similar compositional discrepancies (11).

Despite the different methodology used for subtyping, all studies have consistently shown that the gene expression profile of LAR tumors is unique compared to other TNBC subtypes. The LAR subtype represents ∼15–20% of TNBC. It is characterized by androgen receptor (AR) protein expression and androgen-induced in vitro growth stimulation that can be inhibited using AR-targeted approaches (6,7). Despite evidence for AR signaling in vitro, clinical benefit from single-agent AR-targeted therapies in TNBC has been limited. Studies evaluating bicalutamide, enzalutamide, and abiraterone have reported median progression-free survival ranging between 2.8 and 3.3 months, and response rates measured in single digits (0–8%) (17–19). Of note, these studies used immunohistochemical (IHC) detection of nuclear AR protein expression, with cut-points of >0% (19) or ≥10% expression (17,18) to identify patients for treatment. Although recent advances have been made in understanding the LAR subtype using clustering methods, no LAR-specific molecular signature has demonstrated clinical utility.

The tumor microenvironment (TME) is a complex entity that includes the surrounding stromal tissue, arteriole support and a heterogeneous population of immunological components (20). Recent modeling efforts using single-cell and fluorescent activated cell sorting have been developed to deconvolve the bulk tissue heterogeneity (20–23). Interest has been the immune contribution as its central to carcinogenesis and progression of the disease (20,24–26). Generally, breast cancers are considered less immunogenic than other cancer types (24). However, among the clinical subtypes, TNBCs are the most immunogenic and exhibit higher tumor-infiltrating lymphocytes (TILs), enriched in CD8+ T cells and immunosuppressive FOXP3+ regulatory T cells (20). In TNBC, the addition of immunotherapy (e.g. pembrolizumab) to cytotoxic chemotherapy has resulted in improvements in progression-free and overall survival in the metastatic setting (among PD-L1-positive tumors) (27,28) and improvements in pathologic complete response and event-free survival in non-metastatic TNBC (regardless of PD-L1 expression) (29). The immunological differences among the TNBC subtypes, especially between LAR and non-LAR subtypes, need further investigation.

## MATERIALS AND METHODS

### LAR-Sig signature development

To develop the LAR signature, we used data from 123 female patients with non-metastatic TNBC disease obtained from The Cancer Genome Atlas (TCGA) data portal. Eligibility criteria used to select these samples and detailed bioinformatics methods are provided in the Supplementary Materials. Data were normalized with conditional quantile normalization (CQN v1.8.0) (30). We identified the best k-means clustering partition of the LAR cohort within the TCGA RNASeq data using AR correlated genes (31). Subsequently, we developed a centroid model on the 426 differentially expressed genes. Additional methods can be found in the Supplementary Materials.

### LAR-Sig signature benchmarking

We benchmarked our LAR-Sig model's performance using seven independent datasets (Table 1) and compared it with the three published TNBC subtyping methodologies: TNBCtype-4 (15), fuzzy clustering (5,32) and non-negative matrix factorization (NMF) (11,33). Three microarray experiments (GSE25055, GSE25065, GSE32646) used the Affymetrix 133a platforms, while the fourth (GSE106977) used the Affymetrix HTA 2.0 array. A batch effect was observed only in GSE32646, which was accounted for using the combat algorithm from the sva package (34). Three of the TNBC datasets on which our model was benchmarked were derived from RNA sequencing platforms, including BrighTNess, CALGB 40603, and BEAUTY studies (35–37). Clinical TNBCs (IHC <2 or FISH ratio of <2.0, if IHC >1) were identified and selected from the non-TNBC trials (BEAUTY, CALGB 40603, GSE25055, GSE25065 and GSE32646) (38,39). Concordance was evaluated using kappa statistics obtained from the IRR package (version 0.84.1) (40).

**Table 1.** Testing datasets. We utilized datasets from the Affymetrix microarray platform (hgu133a and HTA array) and RNA-Seq data

ID	Dataset	*n*	TNBC	Platform
A	GSE106977	119	119	Affymetrix HTA 2.0
B	GSE25055	310	116	Affymetrix U133A Array
C	GSE26055	198	60	Affymetrix U133A Array
D	GSE32646	115	26	Affymetrix U133A Array
E	BrighTNess	482	482	RNASeq
F	CALGB 40603	389	241	RNASeq
G	BEAUTY	126^a^	42	RNASeq

^a^ Additional RNASeq samples were available, whereas the original manuscript reported samples having both RNASeq and DNASeq data.

### Evaluation of response to neoadjuvant chemotherapy

Additionally, we identified TNBC patients from seven independent gene datasets who received NAC to investigate whether the response to NAC differed between LAR and non-LAR TNBCs (Supplementary Materials). The TNBC samples were selected (see Signature benchmarking above) based on their published hormonal receptor status and HER2 expression status. For the GEICAM/2006-03 clinical trial, GSE106977, the identification of TNBC samples was provided (41). Pathological complete response (pCR) designation was retrieved from supplementary materials of manuscripts accompanying the four GSE datasets (38,39,41,42) and from patient-level data from the BEAUTY study (43). Patient-level data for BrighTNess and CALGB 40603 was provided through the *Alliance* Standardized Translational ‘Omics Resource (ASTOR) via Alliance for *Clinical Trials* in Oncology. Among these patient cohorts, there were 13 treatment arms. We performed the meta-analysis adopting a random-effects model across the thirteen treatment arms to derive the across-study odds ratio (44), using the SAS 9.4 procedures/tools NLMIX (45) and GENMOD (46).

### Deconvolution of bulk sequencing data using single-cell TNBC dataset

Twenty cell types were previously observed in a single-cell RNA sequencing experiment, originating from five patients with primary TNBC disease (47). We obtained the single-cell gene expression counts and t-SNE clustering scheme and constructed a balanced dataset with prevalent and low abundance cell types. The balanced dataset consisted of eighty nearest neighbors to each of the twenty centroids to adequately represent the least prevalent cell type (i.e. 106 immature perivascular-like fibroblasts, imPVL). The mean-dropout relationship (high zero counts while maintaining a high mean expression level) was evaluated, and the features were reduced to 3205 genes (*a* = 1.5, *b* = 1.1) (48). A deconvolution model was then constructed using the balanced TNBC single-cell data and CIBERSORTx (49,50) method. We combined and scaled the seven NAC datasets (1086) along with the TCGA (*n* = 123) dataset using the SVA package and 9050 common gene features (by gene symbol, 1209 samples) (34). Linear model analysis was performed on each cell type using limma (v 3.46.0).

### Clinicopathological characterization (Mayo-TNBC Cohort)

We subsequently applied the LAR-Sig signature to a cohort of women diagnosed with TNBC at Mayo Clinic between 1 January 1985 and 31 December 2012, treated with upfront surgery (no neoadjuvant therapy). The procedures to assemble this cohort, clinicopathological characteristics and outcomes were reported previously (51). Among the 605 women in this cohort, 269 had adequate tumor tissue for RNA extraction. Sufficient formalin-fixed paraffin-embedded primary breast tissue for RNA extraction and sequencing was available from 269 out of 605 women (51). Additional methods can be found in Supplementary Materials. Fisher exact tests were used to assess whether patient or disease characteristics at diagnosis differed between the LAR and non-LAR groups. Lymphocyte-predominant breast cancer was defined as 50% or more of either stromal or intratumoral lymphocytic infiltration (52). This study was approved by the Mayo Clinic Institutional Review Board, 12-004582. The need for obtaining inform consent was waived by the institutional review board, given that the study was retrospective and non-interventional (51).

### Geneset enrichment and visualizations

Silhouette plots were constructed with the cluster R package, alluvial diagrams were built with the alluvial {v 0.1.2} package (53), and heatmaps were generated with pheatmap (54). We combined and scaled the seven datasets along with the TCGA datasets using the SVA package and 9050 common gene features (by gene symbol) (34). GSVA analysis (55) of the combined dataset was evaluated using genesets from cancerSEA (56) as well as proposed immune genesets (57,58). Parametric gene set enrichment analysis {PGSEA, v 1.36.6} of the LAR-Sig signature, as present in the combined dataset, was performed (59) evaluating the C2 genesets from MsigDB (60).

## RESULTS

The Luminal Androgen Receptor samples have been the most consistently distinct cohort among the TNBC subtypes. We investigated whether a two-cluster model more adequately described TNBC data and developed a signature to distinguish the LAR samples. Further, we investigated whether the tissue heterogeneity observed with the LAR samples influence the NAC response rates.

### Identification of a 426 gene signature between LAR and non-LAR tumors using TCGA cohort

**Figure 1.**
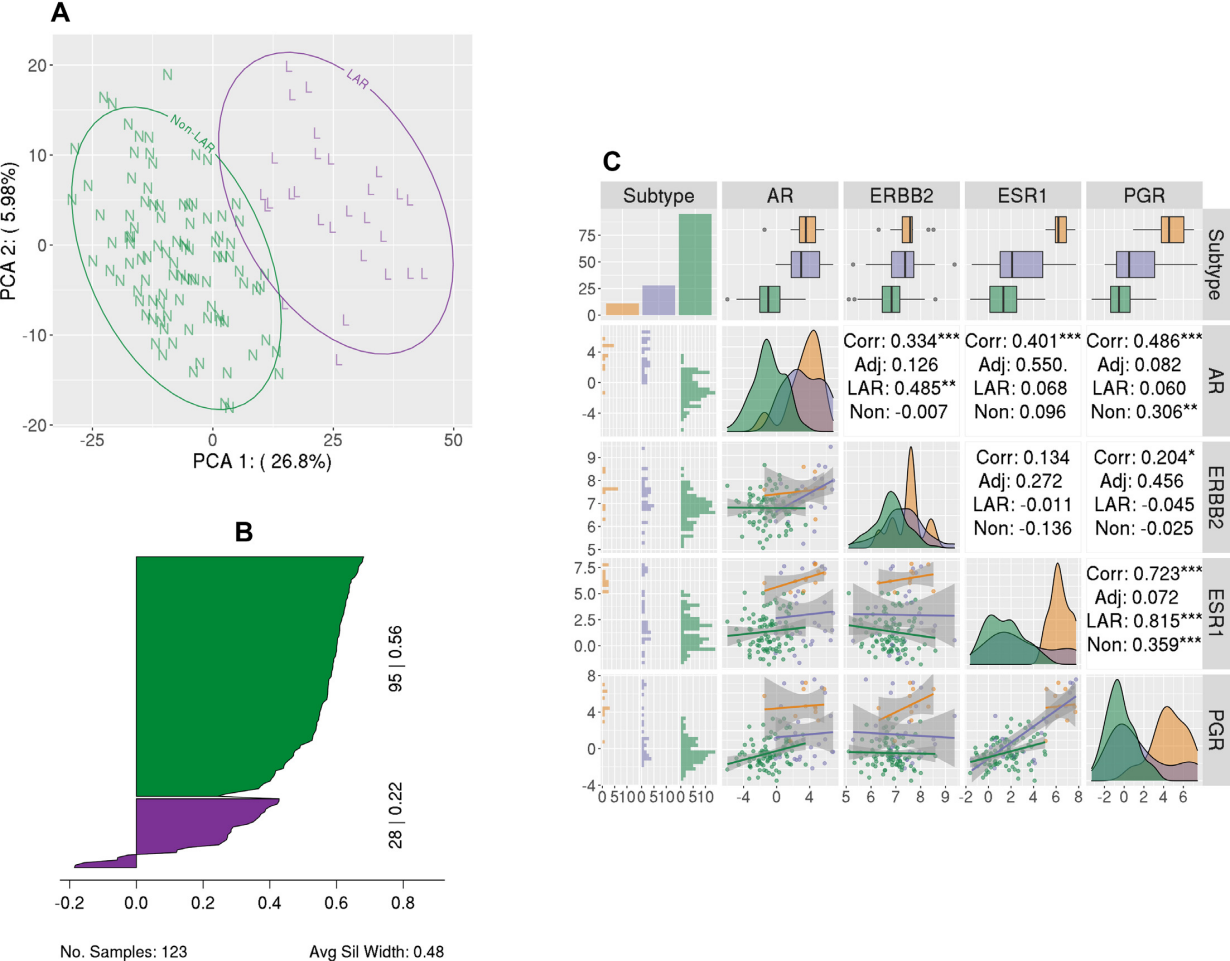
LAR-Sig signature from the TNBC dataset. (**A**) Principal component analysis of the 1067 genes correlated with AR expression, depicting the separation of the LAR cohort. LAR samples are circled and indicated with a purple ‘L’, while non-LAR samples are circled and indicated with a green ‘N’. (**B**) Silhouette plot of the 426 differentially expressed genes (Bonferroni corrected and |logFC| > 2). Clusters have demonstrated significance, with average silhouette width of 0.48. (**C**) A pairs plot depicting the relationship of the expression levels for the HR genes, including AR. We observe that AR expression among the LAR samples is elevated with respect to the non-LAR samples; however, the expression is not increased with respect to adjacent normal tissue from TNBC patients. There is a notable positive correlation between ERBB2 expression and AR expression among the LAR samples, while AR expression has a stronger positive correlation with ESR1 expression among normal adjacent tissues.

We confirmed the appropriateness of the two-cluster approach using *k*-means clustering analysis (*k* evaluated from 2–10) of the 1067 genes correlated with AR expression using the TCGA TNBC samples (ER, PR and HER2 negative). Two optimal clusters were observed based upon the average silhouette width, representing 28 LAR and 95 non-LAR samples. The PCA of the 1067 genes is provided in Figure 1A, where we observed that the first two components explained 32.8% of the variance (26.8% and 6.0%, respectively).

Differential expression analysis of LAR versus non-LAR using the TNBC TCGA cohort was performed with edgeR for the 16,297 genes with a median read count ≥ 32. We observed 4318 genes differentially expressed after correcting for multiple testing errors, using Benjamini and Hochberg correction. Given the large number of differentially expressed genes, we used a more stringent approach - absolute log fold change >2 and *p*-value ≤0.05 after applying Bonferroni's correction to the familywise error rate – to identify the more stringent gene set of 426 genes (Supplementary Table S1), which was subsequently used to construct a shrunken centroid classification model. The average silhouette width for these two clusters was 0.48 (0.22 for LAR and 0.56 for non-LAR), as demonstrated in Figure 1B (Supplementary Table S2). Lastly, we evaluated the expression levels in the LAR, non-LAR samples of the clinically defining hormone receptors, and AR to non-adjacent breast tissue samples from the TCGA. We observe that all four genes' expression is downregulated in the non-LAR cohort (Figure 1C). Moreover, the AR over-expression that characterizes the LAR cohort is not significantly different than that of the non-adjacent tissue.

### Clustering Confirmation of LARs in seven independent transcriptomics NAC datasets

We applied the LAR-Sig to seven independent NAC datasets to confirm its ability to reproduce the LAR designation. These seven datasets are detailed in Table 1 and represent recent clinical trials that have not been interrogated with respect to the LAR subtype. Further, three of the studies characterize the transcriptional profile of TNBC samples using RNA sequencing.

**Figure 2.**
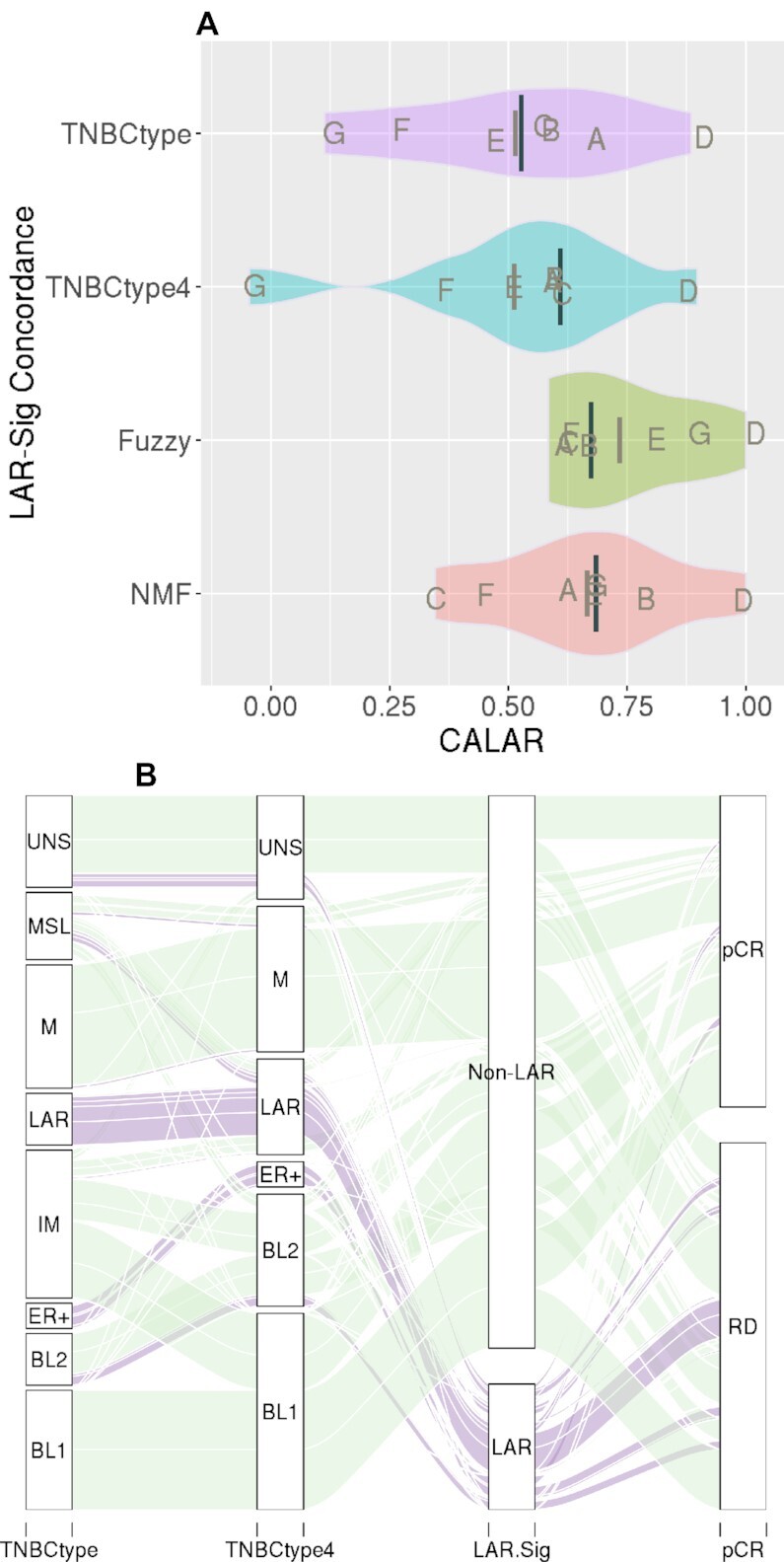
LAR-Sig concordance in LAR identification. (**A**) The distributions of kappa statistics observed between LAR-Sig and four clustering approaches (TNBCtype, TNBCtype-4, NMF, and fuzzy clustering), for the seven NAC datasets. Median kappas is provided as the darker and longer line, with mean kappa statistic provided with a shorter and lighter line. Concordance was observed to be lowest among comparisons to the refined TNBCtype-4 and also observed between the other clustering methods and TNBCtype-4, suggesting overfitting occurred in the initial efforts. (**B**) Alluvial diagram depicting the classification agreement among the original TNBCtype, the refined TNBCtype-4, LAR-Sig and pathological complete response to NAC intervention. We observed a high degree of LAR samples failing to achieve pCR, which was evaluated further.

Clustering solutions are challenged with not having a known benchmark; therefore, benchmarking often involves evaluating the agreement between accepted solutions. We subsequently confirmed that LAR-Sig demonstrated reasonable concordance with the previously proposed clustering methods (TNBCtype, TNBCtype4, Fuzzy and NMF) for these seven datasets. Figure 2A presents the pairwise distributions of the Kappa statistic as violin plots, as observed for the LAR-Sig centroid model and previous clustering approach. The clustering concordance observed for each dataset is indicated with letters (A–G) corresponding to Table 1. We observed the best concordance among the smallest dataset (D, GSE32646), with LAR-Sig demonstrating perfect agreement with the NMF and Fuzzy based approach. The most consistent dataset was D (GSE32646) and the most inconsistent dataset was G (BEAUTY). TNBCtype-4, although a purportedly refined version of TNBCtype demonstrated concordance measures as the original and both were markedly lower in concordances (0.61) than subsequently proposed models. Similarly, the Kappa statistics between TNBCtype-4 and the Fuzzy clustering and NMF clustering approaches were also low: 0.53 and 0.38, respectively. Figure 2B presents the parallel set analysis for the classification of the 1086 samples representing the seven NAC datasets, including the original TNBCtype classifications for historical purposes. We observe that only a few misclassified LAR samples (MSL) are correctly caught with the TNBCtype-4 refinement, while the dismissed unstable sample remains misclassified.

### Neoadjuvant chemotherapy response and TNBC subtype

**Figure 3.**
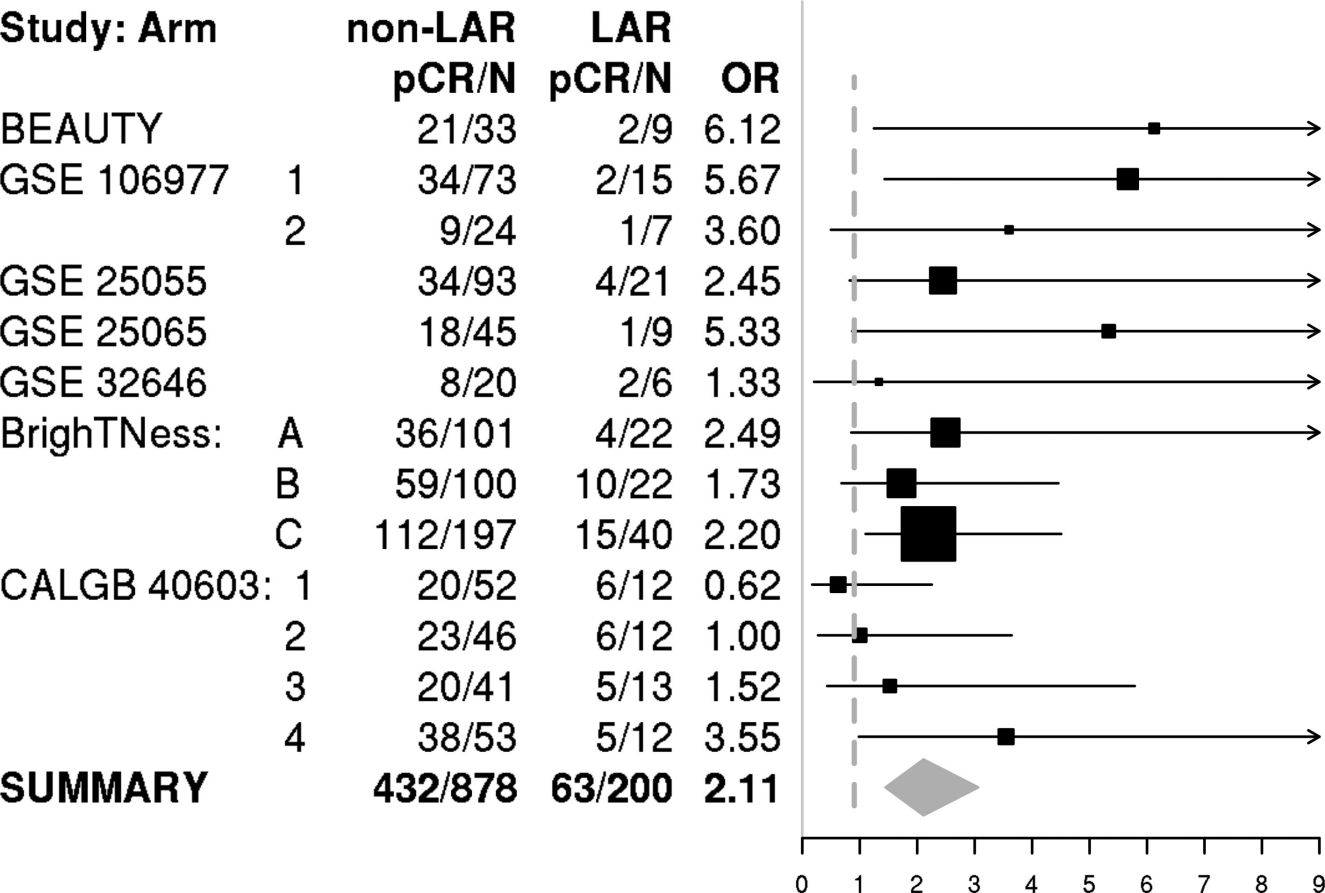
A meta-analysis of NAC treatment response among the LAR and non-LAR TNBC samples. We performed a random-effects model, in SAS 9.4, across the fourteen treatment arms to derive the across-study odds ratio. The sample size within the LAR group was small for many of the NAC treatment arms; however, in 11 of the 13 NAC treatment arms the pCR rate was higher in the non-LAR (35.6% to 71.7%) group compared with the LAR group (13.3–45.5%). The expected or average value [95% CI] of the odds ratio was 2.11 [1.33, 2.89], providing evidence that LAR are significantly less likely to respond to current NAC regimens.

We identified four microarray studies submitted to GEO that included TNBC patients treated with NAC (Table 1), as well as RNA seq data from the BrighTNess, CALGB 40603 studies, and the BEAUTY TNBC subset. Of the 1086 samples, 1078 had pathological response data available. We applied the LAR-Sig centroid model to individual study gene expression datasets and performed a metanalysis of pCR (yes/no) according to the TNBC subtype. The pCR rate among LAR samples was 13.34%, compared to 39.15% in non-LAR samples (Table 2). The results of random-effects modeling indicated that the odds of a pCR was 2.11 (95% CI: 1.33–2.99), more likely for non-LAR TNBC than LAR TNBC (Figure 3). As shown in Table 2 and Figure 3, pCR rates were consistently lower in the LAR group, except for two arms from CALGB 40603 trial where the OR was <1.0 (Arm 1: Taxane followed by A/C) and OR was 1.0 (Arm 3: Taxane & Carboplatin followed by A/C).

**Table 2.** Table 2 shows the pCR rates according to LAR and non-LAR groups for 13 neoadjuvant chemotherapy (NAC) treatment arms across seven clinical trials. Within the LAR group, the pCR response rates ranged from 13.3% to 45.5%, while within the non-LAR group, the pCR rates ranged from 35.6% to 71.7%. The sample size within the LAR group was small for many of the NAC treatment arms; however, in 11 of the 13 NAC treatment arms, the pCR rate was higher in the non-LAR group than in the LAR group. We performed a meta-analysis to summarize the LAR effect across the 13 NAC treatment arms, as measured by the odds ratio [non-LAR vs LAR]. A random-effects logit model was adopted [1]. In the model, the NAC treatment arm effects were treated as random as opposed to fixed to better capture the variability inherent in the system more realistically; furthermore, the model included random LAR effects such that the LAR effect was not constrained to be identical in each trial arm stratum. The odds ratio's expected or average value [95% CI] was 2.11 [1.33, 2.89]. There was strong evidence of a LAR effect such that within the non-LAR group, the odds of achieving a pCR was 2.11 times the odds within the LAR group

					pCR				
Id	Trial	NAC Treatment	Arm	Group	Yes	No	Total	% Yes	Odds Ratio
A	GSE106977	Taxane + AC	A	LAR	2	13	15	13.33%	5.68
				non-LAR	34	39	73	46.58%	
		Taxane + Carboplatin+A/C	B	LAR	1	6	7	14.29%	3.60
				non-LAR	9	15	24	37.50%	
B	GSE25055	Taxane + AC	-	LAR	4	17	21	19.05%	2.45
				non-LAR	34	59	93	36.56%	
C	GSE25065	Taxane + AC	-	LAR	1	8	9	11.11%	2.52
				non-LAR	18	27	45	40.00%	
D	GSE32646	Taxane + FEC	-	LAR	2	4	6	33.33%	1.33
				non-LAR	8	12	20	40.00%	
E	BrightNess	Taxane + Veliparib + Carboplatin+A/C	A	LAR	15	25	40	37.50%	2.20
				non-LAR	112	85	197	56.85%	
	BrightNess	Taxane + Carboplatin + A/C	B	LAR	10	12	22	45.45%	1.73
				non-LAR	59	41	100	59.00%	
	BrightNess	Taxane + AC	C	LAR	4	18	22	18.18%	2.49
				non-LAR	36	65	101	35.64%	
F	CALGB 40603	Taxane + AC	1	LAR	6	6	12	50.00%	0.63
				non-LAR	20	32	52	38.46%	
	CALGB 40603	Taxane + Bevacizumab + A/C	2	LAR	5	8	13	38.46%	1.52
				non-LAR	20	21	41	48.78%	
	CALGB 40603	Taxane + Carboplatin + A/C	3	LAR	6	6	12	50.00%	1.00
				non-LAR	23	23	46	50.00%	
	CALGB 40603	Taxane + Carboplatin + Bevacizumab + A/C	4	LAR	5	7	12	41.67%	3.55
				non-LAR	38	15	53	71.70%	
G	Beauty	Taxane + AC	-	LAR	2	7	9	22.22%	6.13
				non-LAR	21	12	33	63.64%	

### Clinicopathological and immunological characteristics of LAR vs. non-LAR within the Mayo-TNBC cohort

Within the Mayo TNBC cohort, 50 (18.6%) of the 269 tumors were molecularly classified as LAR TNBC and 219 (81.4%) as non-LAR by the LAR-Sig signature. We confirmed that patients with LAR TNBC were more likely to be older (*P <* 0.001), post-menopausal (*P* < 0.001), and to have lower grade tumors (*P* < 0.001), low Ki67 proliferative indices ≤15% (*P* = 0.019), and higher median AR expression by IHC (*P* = 0.001). All tumors classified histologically as an apocrine subtype (*n* = 10) were classified as LAR (Table 3A). Additionally, we observed higher regional lymph node rates in the LARs than in the non-LAR samples (45.8% versus 35.2%, *P* = 0.188). Among 267 patients where TIL scoring was available, 66 (24.7%) had lymphocyte-predominant breast cancer. Stromal TIL content tended to be lower in LAR TNBC compared to non-LAR (*P* = 0.083) with significant immunosuppression signaling and decreased levels of key immunological lineages noted among the Mayo LAR subset compared to the non-LAR tumors (Table 3B).

**Table 3A.** Mayo-TNBC cohort: Patient and disease characteristics at initial diagnosis. Table presents and confirms LAR clinical associations of increased age, AR protein expression, and enrichment of apocrine phenotype

Variable	LAR	non-LAR	
	*n* = 50	*n* = 219	*P* value
Age at diagnosis			**<0.001**
<50 years	7 (14.0%)	89 (40.6%)	
50–6, 9 years	26 (52.0%)	102 (46.6%)	
≥70 years	17 (34.0%)	28 (12.8%)	
Menopausal status			
Premenopausal	8 (16%)	102 (46.6%)	**<0.001**
Post-menopausal	42 (84%)	117 (53.4%)	
Histology			
Ca w/ apocrine differentiation	10 (20.0%)	0 (0%)	**<0.001**
Ca w/ medullary features	7 (14.0%)	49 (18.2%)	
Metaplastic carcinoma NST	5 (10.0%)	12 (4.5%)	
Invasive carcinoma NST	28 (56.0%)	158 (58.7%)	
Nottingham grade			
1–2	16 (32%)	12 (6%)	**<0.001**
3	34 (68%)	207 (95%)	
Ki67			
≤15%	17 (34.7%)	39 (18.1%)	
>15%	32 (65.3%)	176 (81.9%)	
(not obtained)	(1)	(4)	**0.019**
AR IHC			
Median	70%	0%	**<0.001**
25th–75th percentile	22.5–92.5%	0–0%	
(not obtained)	(10)	(46)	
AR			
0%	7 (17.5%)	135 (78.6%)	**<0.001**
≥1%	33 (82.5%)	37 (21.4%)	
(not obtained)	(10)	(46)	
Tumor size			
≤2.0 cm	20 (40.0%)	109 (49.8%)	
2.1–5.0 cm	26 (52.0%)	96 (35.7%)	0.434
≥5.1 cm	4 (8.0%)	14 (5.2%)	

**Table 3B.** Mayo-TNBC cohort: patient and immunological characteristics at initial diagnosis. It presents the immunological observations at initial diagnosis. Elevated lymphocytic activity was only marginally observed

Variable	LAR	Non-LAR	
	*n* = 50	*n* = 219	*P* value
Stromal TILs			
Median	20%	25%	0.083
25th–75th percentile	10–40%	15–50%	
Lymphocyte-dominant BC			
Yes	8 (16.0%)	58 (26.7%)	0.145
No	42 (84.0%)	159 (73.3%)	
Lymph node involvement			
positive	22 (45.8%)	76 (35.2%)	0.188
negative	26 (54.2%)	50 (64.8%)	
(not evaluated – NX)	(2)	(3)	

### LAR gene signature was associated with bone relapse and luminal related signatures

**Figure 4.**
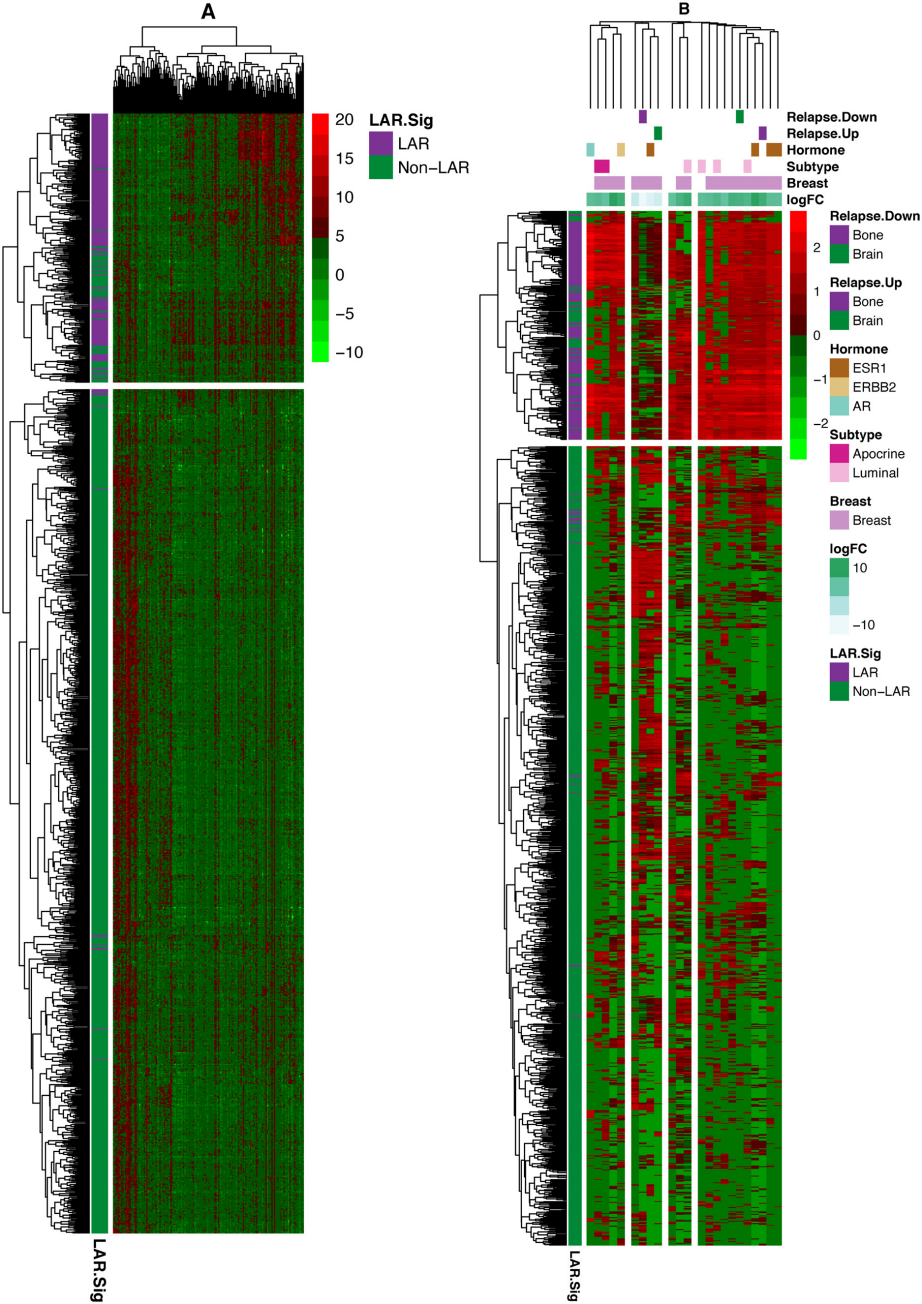
Heatmap (bi-clustering) of the 1209 TNBC samples from TCGA and seven NAC datasets. The samples were ‘batch’ adjusted with the modCombat function in the sva package. The left heatmap presents the bi-clustering performed on the dissimilarity matrix of Pearson's correlation, using complete linkage. Parametric geneset enrichment analysis of these 160 commonly assayed genes between RNA-Seq and microarray from the LAR-Sig signature was performed for the C2 genesets from MsigDB. Genesets were down-selected to the 89 genesets sharing at least 5% (eight genes) of our LAR-signature, while smaller than 600 genes overall were evaluated. Twenty-three genesets were observed to be significantly altered after Bonferroni correction for the family-wise error rate and possessing an absolute log FC of five or more. Using Jaccard's dissimilarity measure, these genesets were clustered based upon their inherent gene membership, and log FC are depicted in the column annotation. Breast cancer signatures predominated the gene signatures (indicated with lavendar). LAR expression positively correlates with bone relapse signatures (marked with purple coloring) from Smid *et al.* While negatively correlated relationships were observed among a similar brain relapse genesets (indicated in green). Here up-regulated genesets are associated with bone relapse up-signature and vice versa, suggesting bone relapse would be more prevalent among LAR samples. The direct relationships also were mostly observed among Smid *et al.*, signatures for Luminal (light pink) and apocrine (red) expression signatures, and AR (light blue-green), ESR1 (brown) and ERBB2 (tan) hormone expression.

Geneset enrichment was performed on the combined and scaled datasets, including the TCGA data. Principal component analysis of the combined data was performed, and any confounding effects of data generation appear to have been negated (see Supplementary File Figures S1–S3). Parametric GeneSet Enrichment Analysis (PGSEA) was implemented to assess 6290 C2 (curated) gene signatures within the MolSigDB. An annotated heatmap of the evaluated genes signature is provided in Figure 4A. The C2 genesets were filtered down to 107 genesets that contained at least 5% of our gene signature while containing less than 600 genes. The resulting genesets were generally differentially expressed (89, 83.2%, Supplementary Table S3) and robust to Bonferroni correction of the family-wise error rate (72 of 89, 80.9%). The inclusion of an absolute fold change greater than 5.0 reduced the space to 23 genesets of interest (Supplementary Table S4, Supplementary File Figure S4). These genesets are provided in Figure 4B, where we have clustered the genesets based upon their Jaccard dissimilarity measure. We observed that the majority of gene signatures identified interrogated breast cancer data (purplish pink). We also observed subtype-specific signatures, including the up-regulation of two signatures associated with apocrine histology and four luminal associated signatures (Figure 4B). The LAR samples demonstrated a positive correlation with genes identified as up-regulated with bone (purple) relapse and a similar positive correlation with genes identified as down-regulated with bone relapse signatures. Conversely, LAR samples demonstrated a negative correlation with genes identified as up-regulated with brain (green) relapse and the same negative correlation with genes identified as down-regulated with brain relapse signatures. These findings suggest that patients with LAR TNBC are more likely to relapse to bone and less likely to relapse to the brain. Further, gene signatures linked to AR, ESR1 and ERBB2 signaling were up-regulated among LAR samples (Figure 4B).

**Figure 5.**
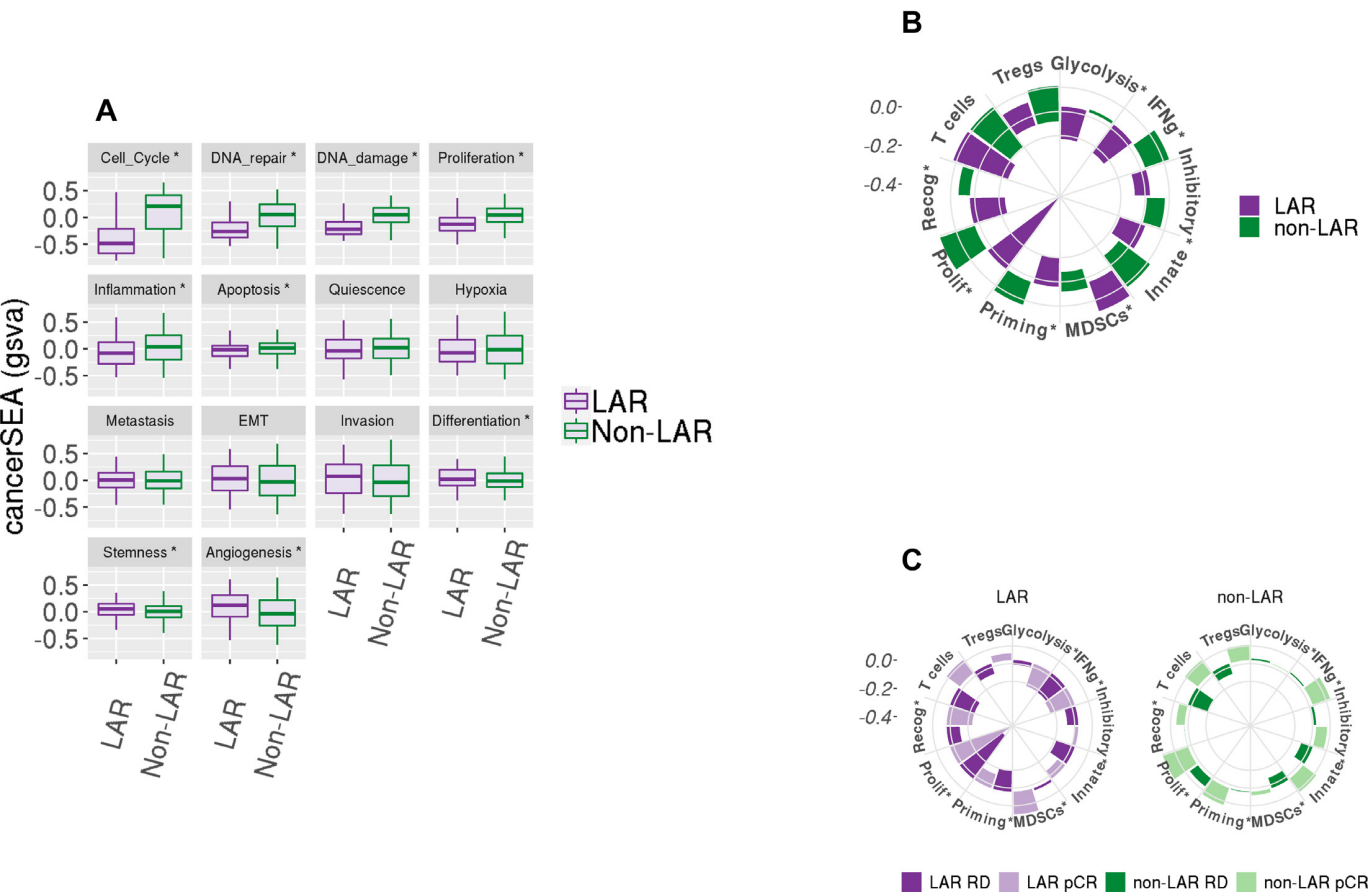
Gene set variation analysis of cancerSEA and Immunograms. Geneset variation analysis (gsva) was performed on the combined and scaled cohort using genesets from the cancer single-cell atlas (cancerSEA) and a recent aggregation of immune gene signatures. (**A**) Ten of the 14 cancerSEA geneset were differentially scored between the LAR and non-LAR cohorts. The down regulation of the cell cycle, DNA damage, and repair associated genes was observed in LARs. The downregulation of the genes associated with these cellular functions may explain the reduced efficacy observed among these LAR NAC samples. (**B**) Similarly, we evaluated recently proposed immune gene signatures and presented them as immunograms. Seven of the ten immune signatures were observed to be differentially scored, indicated with ‘*’. The proliferation signature confirmed the higher expression in proliferation-associated genes among the non-LAR, also observed in the cancerSEA. We also observed a decrease in immunological activity among the LAR, including metabolic glycolysis required to drive innate and acquired immunity, while inhibitory molecules and inhibitory myeloid-derived suppressor cells (MDSCs) mast cell were significantly up-regulated. Regulatory T-cells were also markedly up-regulated, although not significantly. (**C**) Evaluating the same signatures with respect to NAC therapeutic response (1078 samples – 8 samples with missing data) was then performed. No significant immunological activity was observed among the LAR responders. In contrast, the non-LAR responders demonstrated consistent global elevation of immunological expression levels.

Additionally, we performed individual-pathways analysis (using GSVA on fourteen cancer-specific genesets, the cancerSEA) to identify cancer-specific pathway differences among the LAR and non-LAR subtypes (Supplementary Table S5 and Supplementary File Figure S5). Nine of the fourteen genesets demonstrated differences in average scoring means (alpha 0.05), presented in Figure 5A. Particularly, gene signatures related to cell cycle control, DNA damage, and repair were down-regulated (*P* values of 4.56 × 10^–63^, 8.53 × 10^–58^ and 9.24 × 10^–42,^ respectively) in LARs compared to non-LARs. Signatures related to stemness, angiogenesis, and differentiation were up-regulated in the LAR samples relative to non-LAR samples (*P* values of 7.23 × 10^–5^, 7.53 × 10^–8^ and 9.47 × 10^–4^, respectively).

Next, we investigated ten immune signatures recently proposed as potential proxies of specific immunological activities (Supplementary Table S6 and Supplementary File Figure S6). We present the median scores as radial bar plots, providing a cohort-level immunogram (see Figure 5B). We observed a marked decrease in tumor proliferation signature (*P* = 8.28 × 10^–54^) among the LAR relative to the non-LAR. More importantly, we found decreased immunological capacity with decreased glycolysis-driven energetics (*P* = 3.99 × 10^–3^). Additionally, myeloid-derived suppressor cells (MDSCs, *P* = 5.33 × 10^–4^) were activated, while priming and activation of immune cells (*P* = 1.55 × 10^–9^), recognition of tumor cells (*P* = 4.49 × 10^–14^) and γ-IFN signaling (*P* = 3.56 × 10^–5^) were all down-regulated among the LAR samples compare to non-LAR samples.

We subsequently investigated the immunological expression signature associations with a therapeutic response (pCR) among the LAR and non-LAR subtypes, Figure 5C (Supplementary Tables 7 and 8). Non-LAR responders demonstrated a near-global increase in immunological expression patterns (range of *P* values was 2.65 × 10^–5^ to 8.07 × 10^–3^) compared to non-LAR non-responders. While the evaluation of the LAR subset identified that LAR tumors that achieved pCR exhibited decreased expression of genes associated with glycolysis (*P* = 3.44 × 10^–3^) and tumor recognition (*P* = 4.82 × 10^–2^), compared to LAR non-responders. Conversely, LAR tumors achieving pCR demonstrated increased expression among genes associated with antibody-driven innate immunity (7.82 × 10^–2^), see Supplementary Table S8.

### Deconvolution of the LAR tumor microenvironment shows significant epithelial, stromal and immunological differences compared to non-LARs

**Figure 6.**
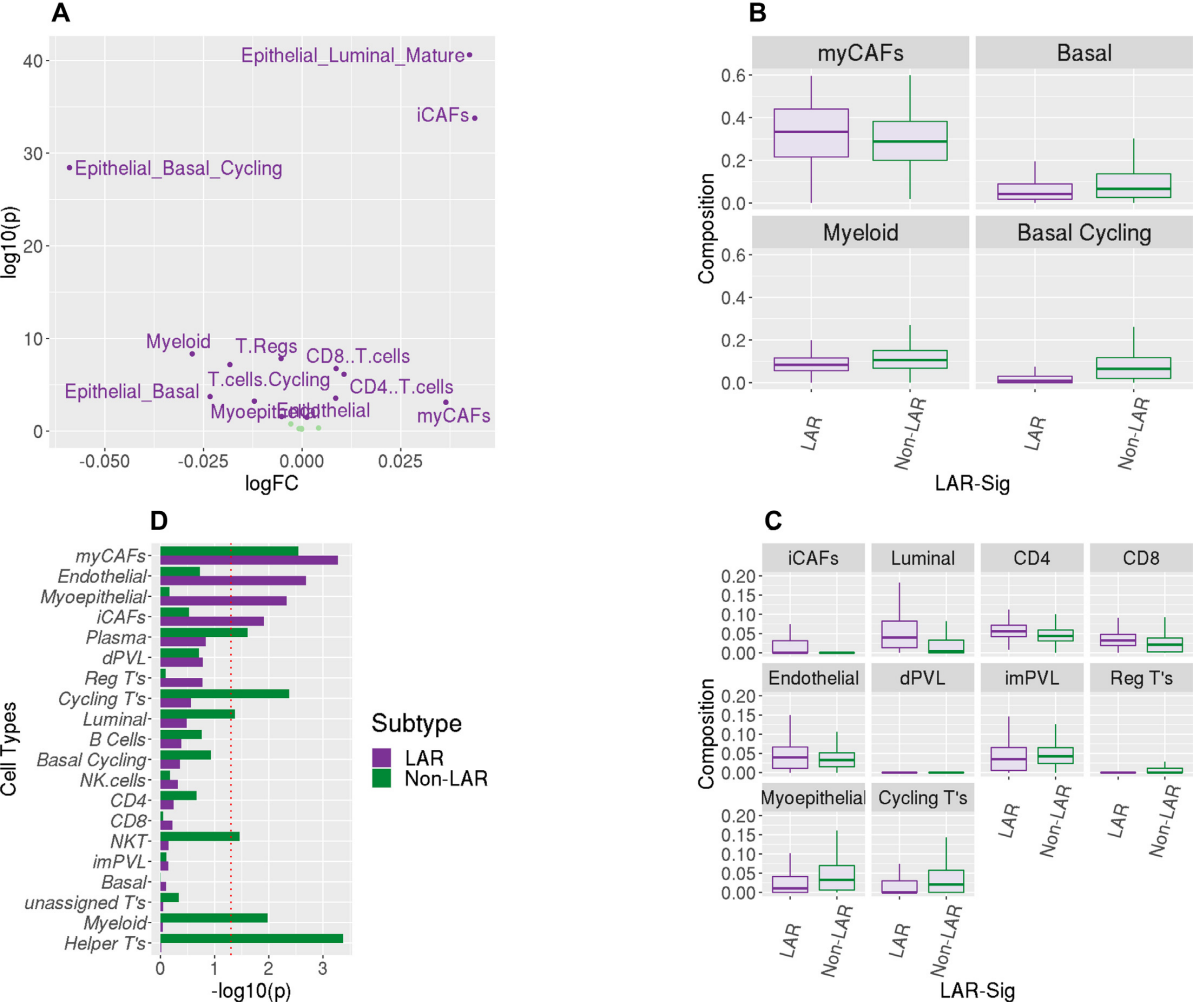
Deconvolution of the RNASeq datasets. TME composition profiles, represented by twenty cell types, were obtained for the three RNASeq datasets. (**A**) Volcano plot shows the observed compositional difference between LAR and non-LAR samples. Linear modeling was performed with the limma package. Fourteen of the twenty cell types were observed to be differently abundant among the TNBC subtypes (α = 0.05). The largest log FC presented in the basal epithelium (cycling) and myofibroblastic cancer-associated fibroblasts (myCAFs). (**B**) We divided these fourteen cell types based on the average prevalence observed in the 1209 samples. We present the distribution of the four most prevalent subtypes (myCAFs, myeloid and two basal epithelia) as boxplots. The basal epithelium was less prevalent in the LAR samples; similarly, there was also a decrease in myeloid cells. In contrast, there was enrichment of myCAFs among the LAR samples. (**C**) We present similar boxplot distributions for the less prevalent cell type distributions, which were observed to be differentially abundant among the subtypes. Similarly, we observed the decreased presence of myoepithelial cells with an increase in mature luminal epithelial cells among the LAR samples. There was a discordant increased presence of CD4+ and CD8+ cells among the LAR, with decreased presence in regulatory and cycling T cells. Minor changes were also observed among the three other fibroblast lineages (inflammatory CAFs and perivascular-like). (**D**) A linear model was also evaluated for compositional associations with the therapeutic response for each of the two subtypes. Here we present the -log10(p value) for the two analyses. A decrease in myofibroblast-like cancer-associated fibroblasts was observed for both subtypes to be associated with pathological complete response. Conversely, LAR samples that responded to treatment also presented an increased presence of inflammatory cancer-associated fibroblasts, as were myoepithelial and endothelial lineages. The response among the non-LAR was associated with increased immunological presence, including myeloid cells, plasma cells, T-helper cells, and cycling T-cells. The cellular increases would appear to be concordant with the immune signature expression patterns we observed in Figure 5C.

The presumption of TNBC subtyping is rooted squarely in the heterogeneity observed among TNBC samples. Differing cellular origins have been proposed in the carcinogenesis of TNBC in contrast to hormone-positive breast cancers. The LAR naming convention itself implies that they are more luminal in origin than basal or myoepithelial. We constructed a 20 cell type deconvolution model containing epithelial (basal, myoepithelial, and luminal), endothelial (arteriole support), stromal (fibroblasts), and immune (T-cells, B-cells, CD4, CD8 and natural killer cells, as well as plasma cells), see Supplementary Table S9). We evaluated the eight datasets (seven datasets listed in Table 1 and the TCGA TNBC cohort) called together (1209 samples) with our deconvolution model to assess the cellular composition of the LAR and non-LAR subtypes. Fourteen of the twenty cell types demonstrated significant changes in abundance among the LAR-and non-LAR subtypes, see Figure 6A. We presented the distributions of these fourteen cell lineages in Figure 6B and C, separated by their score representing their prevalence in the TME. Myofibroblast-like cancer-associated fibroblasts were highly prevalent among both LAR and non-LARs, but with an increased presence in the LAR samples (*P* = 1.30 × 10^–3^), as were the inflammatory cancer-associated fibroblasts (iCAFS, *P* = 1.66 × 10^–34^). In contrast, phenotypic differences between the LAR and non-LAR were minimally explained by the observed epithelial compositional differences. Increased abundances of basal epithelial lineages were observed as expected among the non-LAR samples (basal epithelial, *P* = 4.21 × 10^–4^ and cycling basal epithelial, *P* = 2.40 × 10^–28^), while mature luminal lineages increased among the LAR (*P* = 4.75 × 10^–40^). Moreover, compositional differences in immune abundance may be attributed to decreased immunological expression and the observed decreased rate of NAC response. While CD4+ and CD8+ cells were elevated among the LAR samples (*P* = 7.32 × 10^–7^ and 1.79 × 10^–7^ respectively), cycling (proliferating) and regulatory T cells both were decreased in the LAR versus non-LAR samples (*P* values of 6.71 × 10^–8^ and 5.88 × 10^–8^, respectively).

We subsequently evaluated the composition differences associated with a therapeutic response between responders and non-responders (pCR) for both the LAR and non-LAR cohorts (Supplementary Tables 10–11). Non-LAR responders presented with an increased abundance of natural killer cells, T_helper_ cells, cycling T-cells, myeloid, and plasma cells (*P* values between 0.025 and 0.035), compared to the non-LAR non-responders. LAR patients exhibiting pCR demonstrated increased myoepithelial, iCAFS, and endothelial cells (*P* values of 4.67 × 10^–3^, 1.23 × 10^–2^ and 2.07 × 10^–3^, respectively) compared to LAR non-responders, while decreased expression was observed with myCAFs (*P*-value = 1.06 × 10^–2^), See Figure 6D. The data suggest that the stromal microenvironment is integral to the containment of the LAR primary tumor growth and may influence therapeutics response.

## DISCUSSION

We investigated the cohort of TNBC RNA-Seq data provided by TCGA with the hypothesis that the predominant two TNBC subtypes are non-LAR and LARs. This hypothesis was based on the existing TNBC subtyping literature demonstrating that LAR is the only TNBC subtype that consistently exhibited statistically significant dissimilarity to basal-like TNBC (6,9,10). While several subtypes were previously reported, most of these subtypes did not demonstrate statistical significance (6,11,61). Further, we demonstrated an improved average sample similarity when considering a two-class model (Figure 2A). Additionally, the lowest median concordance (0.53) was observed for TNBCtype and the refined TNBCTYPE-4 suggesting that the original methods overfit the number of TNBC subtypes.

The biological and clinical characteristics of the LAR cohort remain of clinical interest. Thus, we sought to investigate clinical covariates in the Mayo TNBC dataset. Clinicopathologically, LAR TNBC exhibited several unique characteristics compared to non-LAR. Patients with LAR TNBC were generally older, more frequently post-menopausal, had lower grade tumors, lower Ki67 and—as expected—higher AR expression by IHC (Table 3A). In addition, we observed that stromal TIL content was numerically lower in LAR TNBC compared to the non-LAR (Table 3B). Moreover, we tested the hypothesis that the LAR cohort represented a population possessing a poorer response rate to NAC intervention. Our analysis indicated that the non-LAR samples have a 2-fold increase in the likelihood of responding to NAC (Figure 3, Table 2). Reduced pCR response rates were similarly previously reported among the LAR (21.1%) subtype identified with TNBCtype-4 (62).

LAR samples have been characterized as over-expressing AR, while we demonstrate that AR expression among the LAR fails to surpass the expression levels of TNBC adjacent normal tissue (Figure 1C). The interpretation of AR expression as over-expressed and, therein, a potential cancer driver appears to be more of a type-IV interpretation error (63). The more appropriate description of AR would be the characterization of the non-LAR as quadruple negative breast cancer (QNBC) (64). The enrichment of apocrine tumors among the Mayo TNBC cohort suggests that the role of AR still warrants further investigation (Table 3A). The implication of AR has drawn comparisons with prostate cancer and therein the potential role of AR splice variants as well as pioneering transcriptional regulatory roles (65–67). We investigated the overlap of our gene signature with published AR activity signatures and observed minimal overlap (68–73). In contrast, we observed significant overlap with ESR1 gene modules (74,75). Specifically, with the existing ERBB2 (17.9%) and oncotypeDX (14.3%) signatures compared to other AR and ESR1 gene modules (76). Transcriptional differences were observed between the LAR and non-LAR cohorts. The LAR cohort demonstrated expression patterns suggesting that relapses would more likely occur within the bone rather than in the brain, as characterized by signatures reported by Smid et al (77). These findings support previous observations that LAR tumors would be more prone to bone relapse (8). The LAR tumors demonstrate similarities with age, expression levels, and relapse with hormone-positive cancers. A retrospective analysis of 92 TNBC cases identified differences in 22% of the histological assessments. Thirteen discrepancies involved either ER or PGR positivity, while 6 cases involved HER2 designations. While the false negatives did not significantly impact patient treatment, the authors also note that low-positive ER/PGR share similarities with HR- tumors (78). We would be remiss if we did not suggest that the LAR samples appear to be low-positive tumors, needing a histological re-assessment.

While the Subsequent evaluation of cancer-associated genesets (cancerSEA) identified DNA damage and repair and cell cycle control and proliferation as down-regulated among the LAR samples, Figure 5A (Supplementary Table S8, Supplementary File Figure S5). These results suggest that targeted inhibition of such genes within these pathways would be therapeutically ineffective. Decreased proliferation was also observed in the immunogram among the LAR in comparison to the non-LARs, Figure 5B (Supplementary Tables S9–S11 and Supplementary File Figure S6). These observations are in corroboration with previous data demonstrating a lower Ki-67 proliferation index in LAR TNBC (79). Similarly, the immunological molecular activity levels were markedly different and substantially decreased among LAR samples, in contrast to the histological observations (Table 3B).

Compositional differences of LAR responders suggest that primary tumor growth and containment by the myoepithelial, iCAF, and endothelial were associated with influencing the therapeutic response, see Figure 6C (Supplementary Tables 10–11 and Supplementary File Figure S6). Increased metastasis and invasiveness of the LAR disease (Figure 5A) may be associated with the trending increase in lymph node involvement, which we observed among the Mayo-TNBC cohort (Table 3B). Myofibroblast-like cancer-associated fibroblast cells were prominent in both TNBC subtypes, and both subtypes were associated with response. Modulating these tumor-promoting cells could serve as a therapeutic strategy regardless of TNBC subtype stratification and should be further investigated. Moreover, the immunological molecular activity levels were observed to be markedly different. Among those non-LAR TNBC, immunological expression levels are elevated and were more prominently associated with therapeutic response. The developed deconvolution model implicated NKT, cycling, and helper T cells associated with NAC response among the non-LAR, see Figures 6D and 5C. Whereas immunosuppression appears to be nearly universal among the LAR, it may potentially be acquired through or results from the down-regulation of glycolysis (Figure 5C) (80). The data could suggest that the spectrum, strength, and type of immune responses elicited in TNBC disease should be explored further, particularly with respect to treatment strategies, as improved understanding of LAR TNBC will lead to enhanced therapeutic efficacy.

## DATA AVAILABILITY

Data analyzed in this study were a re-analysis of existing data which are openly available at NCBI’s Gene Omnibus. Details for the Alliance clinical trials (CALGB40603 and BrighTNess) and data are available from Ebonie Hatfield at Alliance Translational Research Program. Additional information available in Table 1 and also upon request.

## Supplementary Material

zcac018_Supplemental_Files
