# Supplementary material for: Luminal androgen receptor breast cancer subtype and investigation of the microenvironment and neoadjuvant chemotherapy response
Source: NAR Cancer. 2022 Jun 17;4(2):zcac018. doi: 10.1093/narcan/zcac018 (PMC9204893; doi:10.1093/narcan/zcac018)
Supplement: zcac018_Supplemental_Files [file zcac018_supplemental_files.zip › SupplementaryMaterials_04292022.pdf]

TNBC Materials and Methods

Data

The Cancer Genome Atlas (TCGA)

Clinical characteristics of breast cancer samples were obtained from the TCGA data portal (November 11, 2013, version). Samples from subjects who were male, had metastatic disease, had a prior history of breast cancer, received neoadjuvant therapy, or lacked (documented) interrogation of HER2 status were eliminated from the analysis cohort. Among the 792 breast cancer samples, there were 130 TNBC (ER-, HER2-, and PR- using clinical data) samples. Twelve TNBC samples had paired non-cancerous adjacent tissue samples available for analysis. Breast cancer fastq files were obtained from TCGA, aligned using Tophat {v1.3}, and gene counts were summarized with HTSeq (v0.5.3p3) [1,2]. Conditional quantile normalization (cqn v1.8.0) was performed to adjust for GC bias and gene length [3]. Outliers were identified using normalization stress metrics (with samples exceeding a stress measure above 1.0 or dfArray above 1.5 removed [4]) and principal component analysis (PCA). The final analysis cohort consisted of 123 triple negative samples.

Mayo Clinic TNBC cohort analyses

From a cohort of 9,982 women treated with primary surgery at Mayo Clinic, Rochester, MN between January 1985 and December 2012, we identified 1,156 patients with clinical ER-negative or low (≤10%) and clinical HER2-negative disease. We centrally assessed ER/PR/HER2 staining and HER2 FISH (in cases of HER2 IHC of 2+). A total of 605 patients with centrally confirmed TNBC (defined as ER/PR <1% and HER2-negative per the 2013

ASCO/CAP guidelines) were identified, with clinicopathologic variables previously reported by our group[5].

RNA from 304 FFPE tumor specimens belonging to patients in this cohort was extracted using the HighPure RNA extraction kit and subjected to TruSeq RNA Access library preparation and sequencing on a HiSeq2500. Outliers were examined by looking at expression profiles for PPIA and SF3A1, which have been documented as reliable house-keeping genes to serve as references in FFPE samples [6]. Additional outliers were identified using the normalization stress criteria of 1.1 and dfArray criteria of 1.6 [4]. Adequate RNA was available for 269 tumors and SVA combat algorithm applied to samples from the same flow cell to correct for batch biases [7]. We classified tumors as LAR or Non-Lar using the CABAL signature. Additionally, we assessed AR protein expression by IHC using clone EPR1535(2), Abcam, in 213/269 tumors. The impact of various parameters on RFS and OS was assessed using Cox proportional hazards models.

TNBC Subtyping

We identified the TCGA LAR cohort using K-means clustering analysis of the subset of genes correlated with AR expression. The subset of 1,067 genes demonstrated an absolute correlation (Spearman) greater than 0.35 with AR gene expression. K-means cluster analysis of the correlation dissimilarity matrix was performed with NbClust {v2. 2.0} [8]. The optimal number of clusters was chosen for 2 through 10 clusters, using the average silhouette width as the performance metric [9]. Differential expression using edgeR { v3.3.8}, with Bonferonni correction implemented to control the familywise error rate of multiple testing [10]. A shrunken centroid

model of the gene signature was implemented with pamr {v1.54.1} [11], using the 426 genes which were differentially expressed at an alpha of 0.05 and possessed a |logFC| of 2.0 or greater. The SVA package was used to scale experimental ‘batch’ differences between the training (TCGA RNA Sequencing) data and test datasets (Affymetrix microarray) [7].

Evaluation of chemotherapy response

These datasets included: a retrospective study of anthracycline and/or taxane efficacy using baseline paraffin embedded tissues (GE106977), assayed by the HTA array (Thermo Fisher Scientific) [12]. A small subset of patients were also treated with carboplatin. This dataset was RMA normalized at the transcript level with a simple mean summarization of duplicated probesets measuring the same gene [13,14]. Additionally, datasets evaluated included those reported in the development of a predicted response model for neoadjuvant taxane and anthracycline based chemotherapy. These datasets, the training (GSE25055) and a test (GSE25065) dataset were both assayed on the Affymetrix Human Genome U133A [15]. Similarly, a dataset assayed on the Affymetrix Human Genome U133 plus 2 array which assessed resistance to neoadjuvant paclitaxel followed by 5-fluorouracil/epirubicin/cyclophosphamide (GSE32646) [16]. The U133 arrays were normalized using fRMA [17], genes assayed by multiple probesets were aggregated using Tukey’s median polish[13]. Additionally we investigated 41 TNBC samples from the BEAUTY study[18] which were assess using the Illumina RNA Seqencing platform

References

1. Anders S, Pyl PT, Huber W: HTSeq - A Python framework to work with high-throughput sequencing data. Bioinformatics, 2014
2. Trapnell C, Pachter L, Salzberg SL: TopHat: discovering splice junctions with RNA-Seq. Bioinformatics 25:1105-11, 2009
3. Hansen KD, Irizarry RA, Wu Z: Removing technical variability in RNA-seq data using conditional quantile normalization. Biostatistics 13:204-16, 2012
4. Mahoney DW, Therneau TM, Anderson SK, et al: Quality assessment metrics for whole genome gene expression profiling of paraffin embedded samples. BMC research notes 6:33, 2013
5. Olson JE, Ryu E, Johnson KJ, et al: The Mayo Clinic Biobank: a building block for individualized medicine. Mayo Clin Proc 88:952-62, 2013
6. Aggerholm-Pedersen N, Safwat A, Baerentzen S, et al: The importance of reference gene analysis of formalin-fixed, paraffin-embedded samples from sarcoma patients - an often underestimated problem. Transl Oncol 7:687-93, 2014
7. Leek JT, Johnson, W. Evan, Parker, Hilary S., Fertig, Elana J., Jaffe, Andrew E., Storey, John D.: sva: Surrogate Variable Analysis, (ed 3.14.0). Vienna, Austria, R Foundation for Statistical Computing, 2015
8. Charrad M, Ghazzali, Nadia, Boiteau, Veronique, and Niknafs, Azam: NbClust: NbClust package for determining the best number of clusters. Vienna, R, 2014
9. Tan P-N, Steinbach, M., Kumar, V.: Cluster Analysis: Basic Concepts and Algorithms, Introduction to Data Mining, Addison-Wesley, 2005, pp 487-53
10. Robinson MD, McCarthy DJ, Smyth GK: edgeR: a Bioconductor package for differential expression analysis of digital gene expression data. Bioinformatics 26:139-40, 2010
11. Hastie T, Tibshirani, R., Narasimhan, Balasubramanian, Chu, Gil: pamr: Pam: prediction analysis for microarrays. Vienna, R, 2013
12. Santonja A, Sanchez-Munoz A, Lluch A, et al: Triple negative breast cancer subtypes and pathologic complete response rate to neoadjuvant chemotherapy. Oncotarget 9:26406-26416, 2018
13. Gautier L, Cope L, Bolstad BM, et al: affy--analysis of Affymetrix GeneChip data at the probe level. Bioinformatics 20:307-15, 2004
14. MacDonald JW: hta20transcriptcluster.db: Affymetrix hta20 annotation data (chip hta20transcriptcluster, (ed 8.7.0). Vienna, Austria, R Foundation for Statistical Computing, 2017
15. Hatzis C, Pusztai L, Valero V, et al: A genomic predictor of response and survival following taxane-anthracycline chemotherapy for invasive breast cancer. JAMA 305:1873-81, 2011
16. Miyake T, Nakayama T, Naoi Y, et al: GSTP1 expression predicts poor pathological complete response to neoadjuvant chemotherapy in ER-negative breast cancer. Cancer Sci 103:913-20, 2012
17. McCall MN, Bolstad BM, Irizarry RA: Frozen robust multiarray analysis (fRMA). Biostatistics 11:242-53, 2010
18. Goetz MP, Kalari KR, Suman VJ, et al: Tumor sequencing and patient-derived xenografts in the neoadjuvant treatment of breast cancer. JNCI: Journal of the National Cancer Institute 109:djw306, 2017

LAR-Sig Manuscript

Supplementary Materials

Supplementary Figure 1

The data from 7 NAC TNBC studies and the TCGA TNBC were scaled together, using the combat function in the sva package. The final dataset contained 9050 genes and 1209 TNBC samples all classified using LAR-sig. A PCA plot is presented here demonstrating here with colors indicating the call from our centroid model and the shapes indicate the studies the samples were derived from. Any study-associated bias appears to have been minimized.

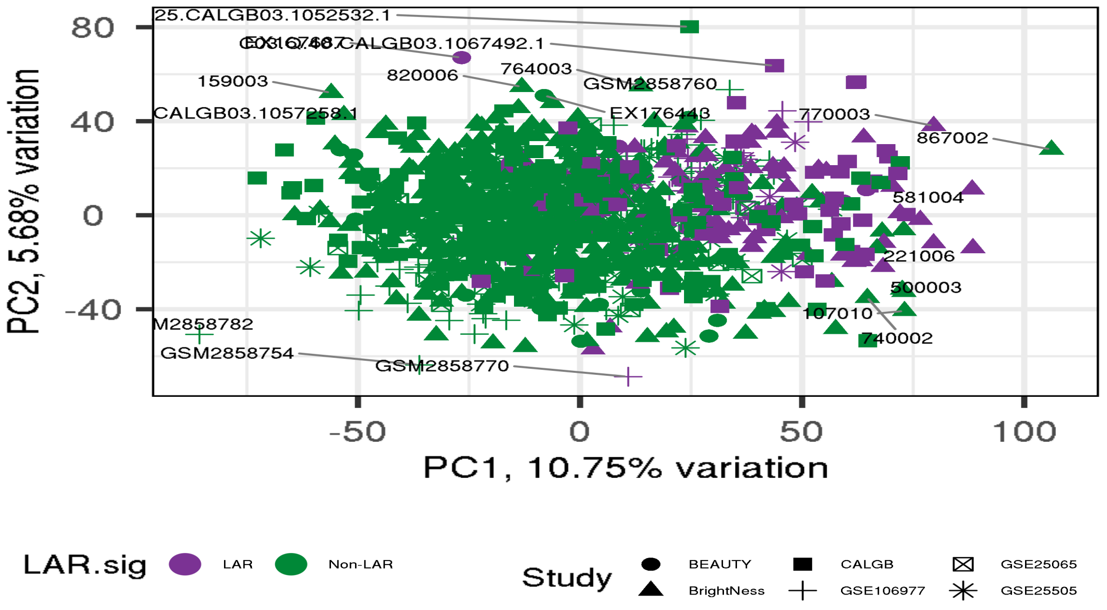

Supplementary Figure 2

An eigen correlation plot from the PCAtools package. The first five PCA eigen values were correlated to the covariates of study and TNBC subtype. The first principal component is strongly correlated with TNBC subtype, and according to the previous plot explains 10.75% of the data variance. The other components also correlated with subtype, and none of which demonstrate notable correlation with study origins.

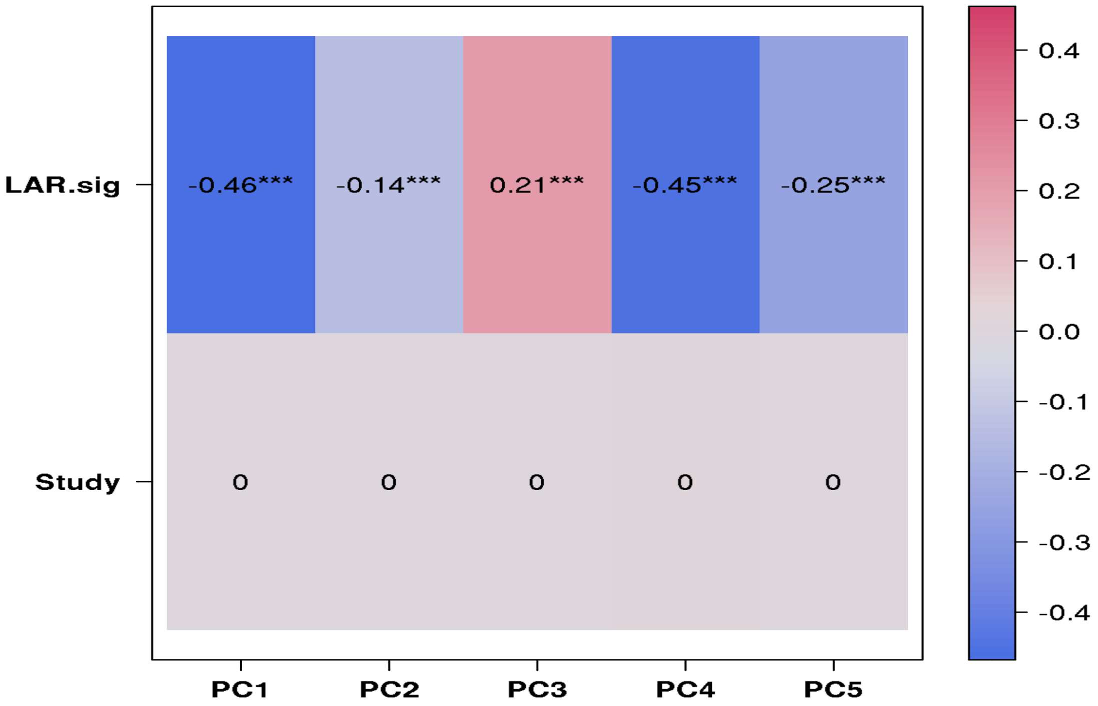

Supplementary Figure 3

Average silhouette width of the scaled data. Average silhouette width was calculated for our models signature genes which were observed in the scaled data (160) and assayed on all the platforms. We also compared using the top 10% of the most variable genes and a similar sized geneset using the top 2% of most variable genes. Our signature genes present with a nearly two fold increase in average silhouette width, which represents the within cluster similarity and between cluster dissimilarity.

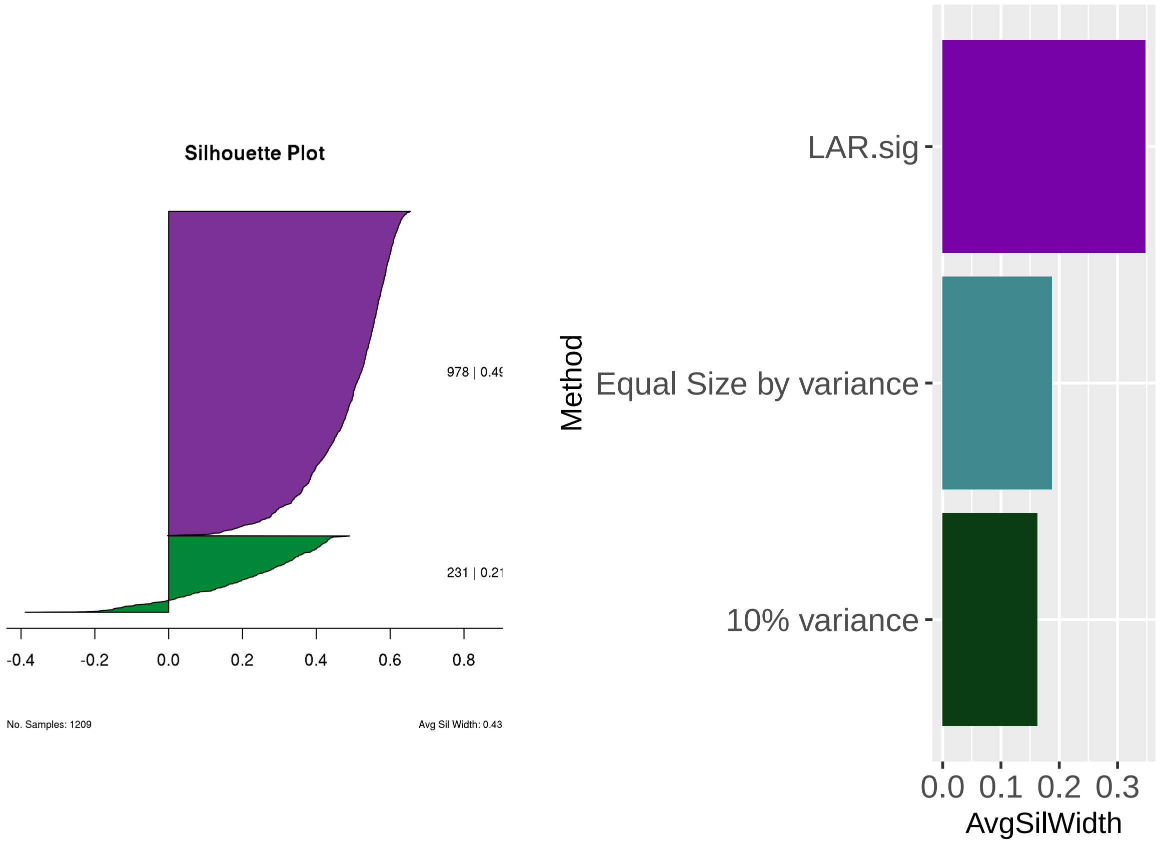

Supplementary Figure 4

Twenty-three genesets observed to be significant with PGSEA (Parametric GeneSet Enrichement Analysis). These genesets were significant after adjusting for the the family wise error rate (Bonferroni) and presented with an absolute logFC of five or more. Genesets were generally associated with luminal observed differences.

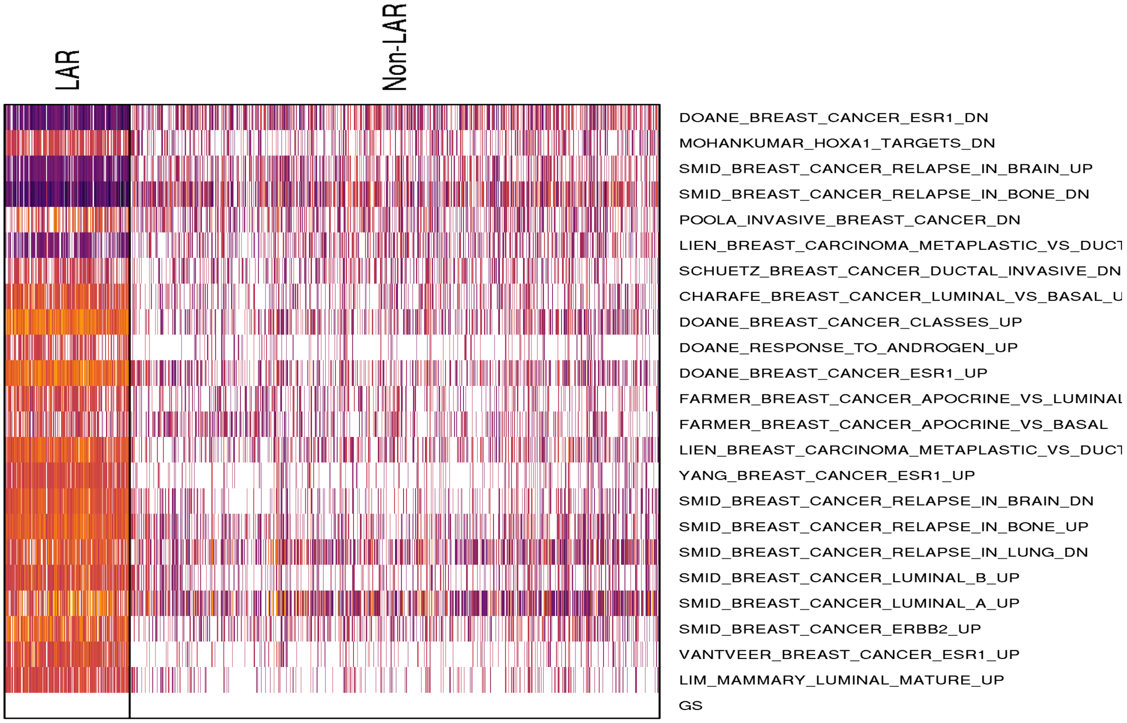

Supplementary Figure 5

Cancer geneset enrichment analysis. Fourteen cancer associated genesets were scored with GSVA (geneset variation analysis). Single samples were evaluated for a difference in means using a linear model (limma). Nine genesets representing cancer processes were observed to present differently among the two TNBC subtypes. Angiogenesis, differentiation, and stemness were associated with increased expression among the LAR subtype.

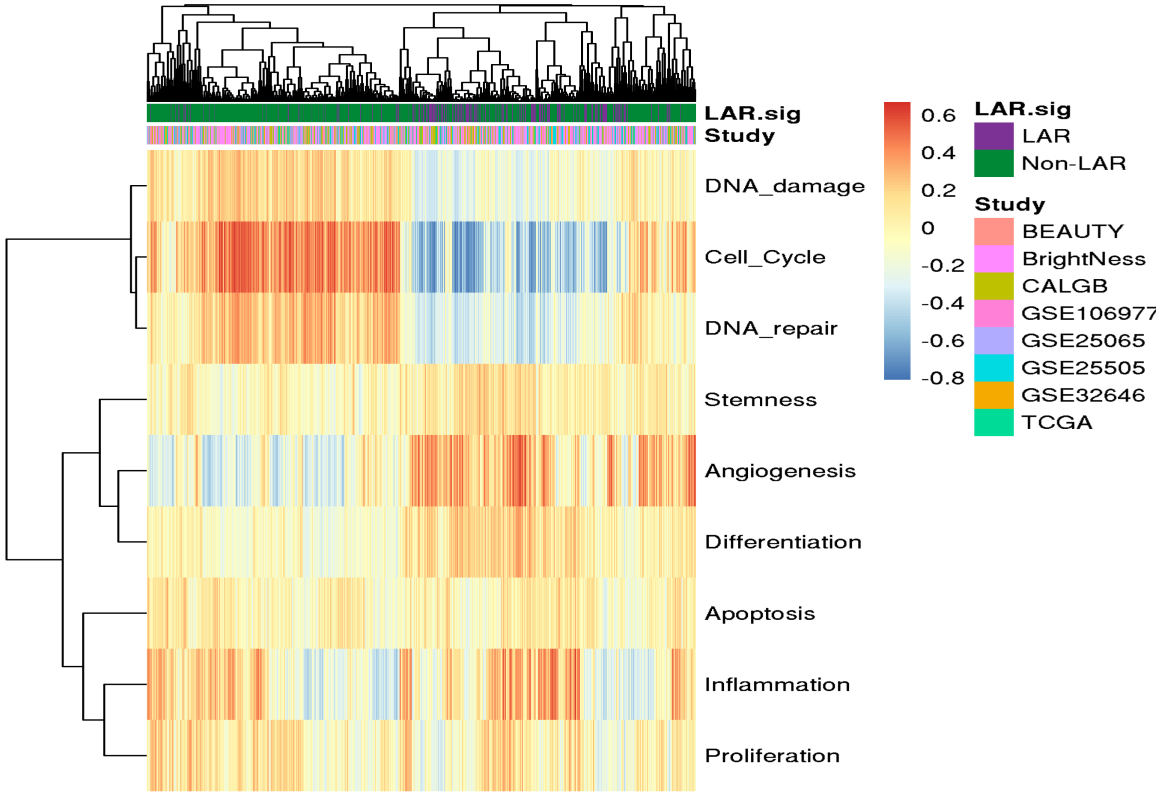

Supplementary Figure 6

Ten immune associated signature recently proposed for immunogram construction were also scored with GSVA. Limma was again used to evaluate for immunological differences. Differencess were observed in 7 of the ten signatures. Interestingly, innate immunity, Inhibitory cells (Tregs), and T cell signaling were not observed to be altered between the subtypes.

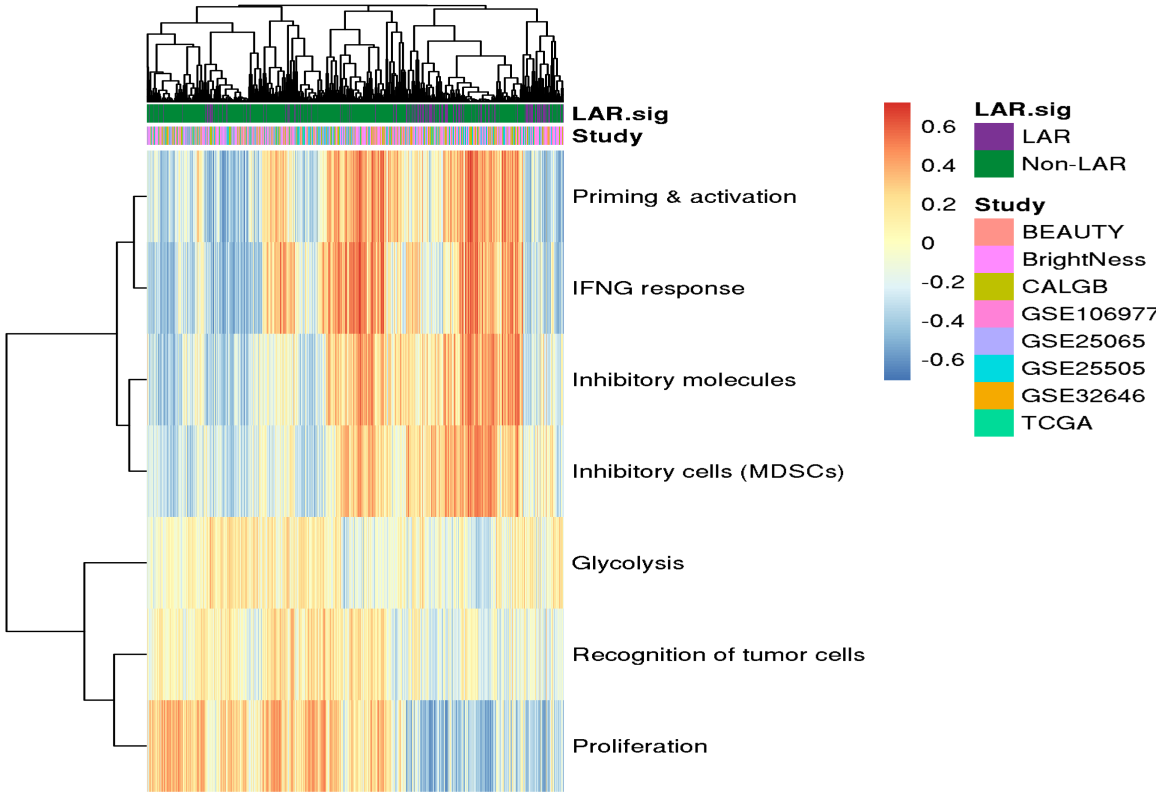

# *Supplementary Table 4* : 23 signifcant C2 genesets of interest							
ID	logFC	AveExpr	t	P.Value	adj.P.Val	B	Bonferroni
DOANE_BREAST_CANCER_ESR1_DN	-9.043	-2.430	-20.085	0.000	0.000	153.540	0.000
MOHANKUMAR_HOXA1_TARGETS_DN	5.422	2.153	15.125	0.000	0.000	86.731	0.000
SMID_BREAST_CANCER_RELAPSE_IN_BRAIN_UP	-7.432	-2.276	-20.236	0.000	0.000	150.169	0.000
SMID_BREAST_CANCER_RELAPSE_IN_BONE_	-11.502	-2.731	-21.466	0.000	0.000	176.490	0.000
POOLA_INVASIVE_BREAST_CANCER_DN	6.608	1.674	12.029	0.000	0.000	56.974	0.000
LIEN_BREAST_CARCINOMA_METAPLASTIC_VS_DUCTAL_UP	-5.969	-1.745	-15.696	0.000	0.000	94.449	0.000
SCHUETZ_BREAST_CANCER_DUCTAL_INVASIVE_DN	5.085	1.424	12.510	0.000	0.000	60.271	0.000
CHARAFE_BREAST_CANCER_LUMINAL_VS_BASAL_UP	8.152	3.669	21.402	0.000	0.000	154.605	0.000
DOANE_BREAST_CANCER_CLASSES_UP	13.186	5.243	31.482	0.000	0.000	288.632	0.000
DOANE_RESPONSE_TO_ANDROGEN_UP	6.112	3.799	14.290	0.000	0.000	67.915	0.000
DOANE_BREAST_CANCER_ESR1_UP	12.849	5.090	29.390	0.000	0.000	265.927	0.000
FARMER_BREAST_CANCER_APOCRINE_VS_LUMINAL	7.061	2.836	18.436	0.000	0.000	118.948	0.000
FARMER_BREAST_CANCER_APOCRINE_VS_BASAL	5.320	1.388	11.445	0.000	0.000	50.819	0.000
LIEN_BREAST_CARCINOMA_METAPLASTIC_VS_DUCTAL_DN	9.673	4.133	23.191	0.000	0.000	182.896	0.000
YANG_BREAST_CANCER_ESR1_UP	6.372	4.747	18.094	0.000	0.000	110.310	0.000
SMID_BREAST_CANCER_RELAPSE_IN_BRAIN_DN	9.171	4.677	24.372	0.000	0.000	186.232	0.000
SMID_BREAST_CANCER_RELAPSE_IN_BONE_UP	10.436	4.242	27.040	0.000	0.000	234.210	0.000
SMID_BREAST_CANCER_RELAPSE_IN_LUNG_DN	8.744	2.649	17.273	0.000	0.000	118.059	0.000
SMID_BREAST_CANCER_LUMINAL_B_UP	7.788	3.858	18.847	0.000	0.000	126.592	0.000
SMID_BREAST_CANCER_LUMINAL_A_UP	10.854	2.673	15.509	0.000	0.000	98.123	0.000
SMID_BREAST_CANCER_ERBB2_UP	10.588	4.332	23.398	0.000	0.000	180.698	0.000
VANTVEER_BREAST_CANCER_ESR1_UP	6.219	5.484	15.125	P.Value	0.000	80.350	Bonferroni
LIM_MAMMARY_LUMINAL_MATURE_UP	6.229	4.174	19.414	0.000	0.000	122.570	0.000

# *Supplementary Table 5* : Results from limma analysis of GSVA of the cancerSEA genesets						
**ID**	**logFC**	**AveExpr**	**t**	**P.Value**	**adj.P.Val**	**B**
Angiogenesis	0.112	0.009	5.404	0.000	0.000	6.554
Apoptosis	-0.044	0.001	-4.115	0.000	0.000	0.536
Cell_Cycle	-0.486	0.009	-17.786	0.000	0.000	132.575
DNA_damage	-0.223	-0.006	-16.916	0.000	0.000	120.487
DNA_repair	-0.249	-0.012	-14.196	0.000	0.000	85.283
Differentiation	0.041	0.011	3.314	0.001	0.001	-2.404
EMT	0.033	0.008	1.421	0.155	0.189	-6.859
Hypoxia	-0.012	-0.004	-0.553	0.580	0.625	-7.714
Inflammation	-0.096	0.010	-4.865	0.000	0.000	3.848
Invasion	0.035	0.004	1.436	0.151	0.189	-6.838
Metastasis	0.004	0.008	0.250	0.802	0.802	-7.836
Proliferation	-0.151	0.010	-11.872	0.000	0.000	58.759
Quiescence	-0.023	0.012	-1.399	0.162	0.189	-6.890
Stemness	0.043	0.010	4.140	0.000	0.000	0.636

# *Supplementary Table 6* : **Results from limma analysis of GSVA of the immunogram genesets**							
**ID**	**Signature**	**logFC**	**AveExpr**	**t**	**P.Value**	**adj.P.Val**	**B**
Innate immunity	LM22_NK_cells_activated	-0.051	0.013	-1.551	0.121	0.151	-6.576
Priming & activation	LM22_Dendritic_cells_activated	-0.156	0.019	-6.087	0.000	0.000	10.453
T cells	LM22_T_cells_CD8	-0.028	0.008	-0.785	0.433	0.433	-7.469
IFNG response	HALLMARK_INTERFERON_GAMMA_RESPONSE	-0.111	0.012	-4.150	0.000	0.000	0.767
Inhibitory molecules	IEGS_immune_escape	-0.074	0.016	-3.206	0.001	0.002	-2.663
Inhibitory cells (Tregs)	LM22_T_cells_regulatory	-0.029	0.017	-0.959	0.338	0.375	-7.316
Inhibitory cells (MDSCs)	Angelova_MDSC	0.075	0.012	3.473	0.001	0.001	-1.780
Recognition of tumor cells	REACTOME_CLASS_I_MHC_MEDIATED_ANTIGEN_PROCESSING_PRESENTATION	-0.111	-0.004	-7.637	0.000	0.000	20.679
Proliferation	REACTOME_DNA_REPLICATION	-0.361	0.000	-16.239	0.000	0.000	111.402
Glycolysis	HALLMARK_GLYCOLYSIS	-0.040	-0.005	-2.885	0.004	0.006	-3.634

# *Supplementary Table 7* : Non-LAR pCR associations with immunogram signatures (GSVA)							
**ID**	**Signature**	**logFC**	**AveExpr**	**t**	**P.Value**	**adj.P.Val**	**B**
Innate immunity	LM22_NK_cells_activated	0.114	-0.033	1.770	0.078	0.255	-4.534
Priming & activation	LM22_Dendritic_cells_activated	0.054	-0.111	1.071	0.286	0.317	-5.461
T cells	LM22_T_cells_CD8	0.106	-0.025	1.499	0.135	0.255	-4.946
IFNG response	HALLMARK_INTERFERON_GAMMA_RESPONSE	0.060	-0.090	1.230	0.220	0.275	-5.289
Inhibitory molecules	IEGS_immune_escape	0.068	-0.045	1.548	0.123	0.255	-4.876
Inhibitory cells (Tregs)	LM22_T_cells_regulatory	0.085	-0.007	1.350	0.179	0.255	-5.145
Inhibitory cells (MDSCs)	Angelova_MDSC	0.060	0.068	1.427	0.155	0.255	-5.045
Recognition of tumor cells	REACTOME_CLASS_I_MHC_MEDIATED_ANTIGEN_PROCESSING_PRESENTATION	-0.064	-0.098	-1.987	0.048	0.241	-4.156
Proliferation	REACTOME_DNA_REPLICATION	-0.014	-0.310	-0.321	0.749	0.749	-5.953
Glycolysis	HALLMARK_GLYCOLYSIS	-0.100	-0.046	-2.960	0.003	0.034	-1.955

# *Supplementary Table 8* : **LAR pCR associations with immunogram signatures (GSVA)**							
**ID**	**Signature**	**logFC**	**AveExpr**	**t**	**P.Value**	**adj.P.Val**	**B**
Innate immunity	LM22_NK_cells_activated	0.108	0.024	3.545	0.000	0.001	-0.342
Priming & activation	LM22_Dendritic_cells_activated	0.083	0.048	3.490	0.001	0.001	-0.530
T cells	LM22_T_cells_CD8	0.118	0.015	3.645	0.000	0.001	0.002
IFNG response	HALLMARK_INTERFERON_GAMMA_RESPONSE	0.091	0.033	3.584	0.000	0.001	-0.209
Inhibitory molecules	IEGS_immune_escape	0.069	0.030	3.190	0.001	0.002	-1.495
Inhibitory cells (Tregs)	LM22_T_cells_regulatory	0.089	0.025	3.136	0.002	0.002	-1.659
Inhibitory cells (MDSCs)	Angelova_MDSC	0.053	0.000	2.655	0.008	0.009	-3.004
Recognition of tumor cells	REACTOME_CLASS_I_MHC_MEDIATED_ANTIGEN_PROCESSING_PRESENTATION	0.050	0.017	3.703	0.000	0.001	0.206
Proliferation	REACTOME_DNA_REPLICATION	0.087	0.067	4.224	0.000	0.000	2.185
Glycolysis	HALLMARK_GLYCOLYSIS	-0.009	0.002	-0.728	0.467	0.467	-6.170

# *Supplementary Table 9* : **TME differences among the LAR and Non-LAR samples**						
**Lineage**	**logFC**	**AveExpr**	**t**	**P.Value**	**adj.P.Val**	**B**
iCAFs	0.044	0.017	12.644	0.000	0.000	66.095
myCAFs	0.036	0.316	3.368	0.001	0.001	-3.383
Plasma_Cells	0.004	0.078	0.738	0.460	0.575	-8.759
Epithelial_Basal	-0.023	0.087	-3.744	0.000	0.000	-2.056
Endothelial	0.008	0.038	3.639	0.000	0.001	-2.443
Myeloid	-0.028	0.110	-5.906	0.000	0.000	8.171
T_cells_unassigned	0.000	0.000	-0.478	0.633	0.633	-8.917
CD8..T.cells	0.009	0.026	5.251	0.000	0.000	4.607
NKT.cells	-0.001	0.016	-0.604	0.546	0.575	-8.849
NK.cells	0.000	0.000	-0.634	0.526	0.575	-8.830
T.cells.Cycling	-0.018	0.036	-5.433	0.000	0.000	5.559
CD4..T.cells	0.011	0.051	4.979	0.000	0.000	3.246
T.Regs	-0.005	0.007	-5.704	0.000	0.000	7.033
Tfh.cells	-0.001	0.013	-0.607	0.544	0.575	-8.847
dPVL	0.001	0.001	2.141	0.033	0.046	-6.743
imPVL	-0.005	0.045	-2.221	0.027	0.041	-6.568
B_Cells	-0.003	0.016	-1.381	0.167	0.223	-8.077
Myoepithelial	-0.012	0.043	-3.449	0.001	0.001	-3.108
Epithelial_Basal_Cycling	-0.059	0.069	-11.511	0.000	0.000	53.879
Epithelial_Luminal_Mature	0.042	0.030	13.994	0.000	0.000	81.776

# *Supplementary Table 10* : TME compositonal differences associated with pCR among the LAR						
**Lineage**	**logFC**	**AveExpr**	**t**	**P.Value**	**adj.P.Val**	**B**
iCAFs	0.035	0.056	2.526	0.012	0.062	-5.367
myCAFs	-0.084	0.344	-3.522	0.001	0.011	-2.477
Plasma_Cells	0.015	0.081	1.459	0.146	0.480	-7.459
Epithelial_Basal	-0.003	0.071	-0.271	0.787	0.926	-8.486
Endothelial	0.017	0.044	3.122	0.002	0.021	-3.744
Myeloid	0.001	0.087	0.118	0.906	0.954	-8.515
T_cells_unassigned	0.000	0.000	-0.135	0.893	0.954	-8.513
CD8..T.cells	-0.002	0.034	-0.525	0.600	0.857	-8.384
NKT.cells	0.001	0.016	0.370	0.712	0.894	-8.454
NK.cells	0.000	0.000	0.709	0.479	0.799	-8.270
T.cells.Cycling	0.005	0.019	1.097	0.274	0.685	-7.920
CD4..T.cells	-0.002	0.060	-0.561	0.575	0.857	-8.364
T.Regs	0.002	0.003	1.383	0.168	0.480	-7.566
Tfh.cells	0.000	0.012	-0.047	0.962	0.962	-8.521
dPVL	0.003	0.002	1.389	0.166	0.480	-7.558
imPVL	0.002	0.040	0.366	0.715	0.894	-8.455
B_Cells	0.003	0.014	0.822	0.412	0.799	-8.184
Myoepithelial	0.021	0.033	2.861	0.005	0.031	-4.493
Epithelial_Basal_Cycling	-0.003	0.020	-0.775	0.439	0.799	-8.221
Epithelial_Luminal_Mature	-0.010	0.064	-0.979	0.329	0.730	-8.042

# *Supplementary Table 11* : TME compositonal differences associated with pCR among the Non-LAR						
**Lineage**	**logFC**	**AveExpr**	**t**	**P.Value**	**adj.P.Val**	**B**
iCAFs	0.002	0.009	1.045	0.297	0.456	-8.574
myCAFs	-0.029	0.309	-2.992	0.003	0.028	-4.662
Plasma_Cells	0.012	0.079	2.251	0.025	0.099	-6.592
Epithelial_Basal	0.000	0.092	0.011	0.991	0.991	-9.120
Endothelial	-0.003	0.037	-1.321	0.187	0.354	-8.247
Myeloid	0.012	0.115	2.563	0.011	0.053	-5.844
T_cells_unassigned	0.000	0.000	-0.740	0.459	0.656	-8.846
CD8..T.cells	0.000	0.024	-0.136	0.892	0.939	-9.110
NKT.cells	0.002	0.017	2.115	0.035	0.116	-6.886
NK.cells	0.000	0.000	0.430	0.668	0.856	-9.027
T.cells.Cycling	0.009	0.040	2.870	0.004	0.028	-5.015
CD4..T.cells	0.003	0.049	1.238	0.216	0.360	-8.354
T.Regs	0.000	0.008	0.250	0.802	0.892	-9.088
Tfh.cells	0.004	0.013	3.536	0.000	0.009	-2.904
dPVL	-0.001	0.001	-1.298	0.195	0.354	-8.277
imPVL	-0.001	0.046	-0.279	0.781	0.892	-9.081
B_Cells	0.003	0.017	1.365	0.173	0.354	-8.188
Myoepithelial	-0.001	0.045	-0.406	0.685	0.856	-9.037
Epithelial_Basal_Cycling	-0.008	0.079	-1.569	0.117	0.292	-7.889
Epithelial_Luminal_Mature	-0.004	0.021	-2.035	0.042	0.120	-7.052
